# The Liverwort and Hornwort Flora of Jeju Island, Republic of Korea: A Volcanic Island with a Unique Mixture of Subtropical, Temperate, Boreal, and Arctomontane Taxa

**DOI:** 10.3390/plants12122384

**Published:** 2023-06-20

**Authors:** Seung Se Choi, Vadim A. Bakalin, Hyun Min Bum, Seung Jin Park, Dae Shin Kim, Ung San Ahn, Myung-Ok Moon

**Affiliations:** 1 Team of National Ecosystem Survey, National Institute of Ecology, Seocheon 33657, Republic of Korea; 2 Botanical Garden-Institute, Far Eastern Branch of the Russian Academy of Sciences, Makovskogo Street 142, 690024 Vladivostok, Russia; 3 Department of Life Science, Jeonbuk National University, Jeonju 54896, Republic of Korea; know8318@jbnu.ac.kr; 4 Division of Botany, Honam National Institute of Biological Resources, Mokposi 58762, Republic of Korea; moss89@hnibr.re.kr; 5 World Heritage Office, Jeju Special Self-Governing Provincial Government, Jeju 63143, Republic of Korea; kds3374@korea.kr (D.S.K.); ungsan@hanmail.net (U.S.A.); 6 Institute of Forestree, Jeju 63133, Republic of Korea; egosari@naver.com

**Keywords:** liverworts, Jeju Island, distribution pattern, subtropical, temperate, nonvascular plants

## Abstract

Jeju Island, due to its position at the southern tip of the Korean Peninsula in Northeast Asia, is a on the unique enclave of the many southern elements in the area and features a mixture of subtropical, temperate, boreal, and arctomontane taxa. Among the arctomontane species recorded in this study was *Anthelia juratzkana*; among the temperate species was *Dactyloradula brunnea*, and subtropical species were *Cavicularia densa*, *Pallavicinia subciliata*, *Wiesnerella denudata*, and *Megaceros flagellaris*. A valuable species as first recorded for the Jeju Island is *Cryptocoleopsis imbricata*. The distribution patterns of these species suggest that the flora of Jeju Island is a meeting place between boreal and subtropical floras. We recorded 222 taxa belonging to 45 families, 80 genera, 209 species, 9 subspecies, and 4 varieties. Among these, 86 species are reported as new to the flora of Jeju Island. A checklist based on a study of 1697 specimens is also provided.

## 1. Introduction

Jeju Island is a volcanic island located between the Korean Peninsula, China, and Japan. Jeju Island was created as volcanic island between about 1.8 million and 1000 years ago, and is either connected to the land (Korean Peninsula) or is located as an island during the glacial and interglacial periods, respectively [1]. Jeju Island is composed of various topographical landscapes, including lava tubes and parasitic volcanoes called oreums. In recognition of the excellence of unique natural environments and diverse species, the large part of Jeju Island was protected as a national park (Mt. Hallasan National Park) in 1970, was further designated as a biosphere reserve in 2002, a World Natural Heritage Site in 2007, and was certified as a Global Geopark in 2010 by UNESCO [2].

A study of bryophytes on Jeju Island was initially conducted by a French Catholic priest and Japanese researchers. Faurie, U. (1847–1915, French Catholic priest) first collected bryophytes from Jeju Island and sent them to European botanists for identification. The specimens were sent to Cardot, if they were mosses, and Stephani, if they were hepatics [3]. Stephani [4] reported several species for Jeju Island, such as *Anthoceros koreanus* Steph. (=*Phaoceros carolinianus* (Michx.) Prosk.), *Solenostoma koreanum* Steph. (=*Protosolenostoma fusiforme* (Steph.) Vilnet and Bakalin), *Jungermannia decurrens* Steph. (=*Solenostoma faurieanum* (Beauverd) R.M. Schust.), and *Lepidozia coreana* Steph. (=*Lepidozia subtransversa* Steph.). Hattori et al. [5] reported 61 taxa, including a new species, *Metacalypogeia quelpartensis* S. Hatt. and Inoue (=*Eocalypogeia quelpaertensis* (S. Hatt. and Inoue) R.M. Schust.).

Hong [6,7], the first Korean researcher, reported 71 taxa based on specimens collected on Jeju Island. Choe [8,9], who comprehensively organized Korean bryophytes, reported 65 taxa based on the literature and collected specimens from Jeju Island. Later, Song and Yamada [10] summarized available information and reported 120 taxa of liverworts on Jeju Island. With a few later additions, 138 liverwort taxa on Jeju Island were recorded in the literature. However, previous studies have investigated and reported only a few points in a short period of time and are insufficient to show the entire flora.

The aim of this study was to investigate the liverwort taxonomic diversity of Jeju Island, to analyze the liverwort distribution patterns across the island, and to understand whether the liverwort flora of the island has peculiar traits. This account describes the obtained results.

## 2. Results

During the field work, 1697 liverwort specimens were collected and identified. A total of 1697 identifications were completed (since the vast majority of specimens contained more than 1 species in the sampled mat). The compiled list of taxa, including data from the literature, comprised 209 species, 9 subspecies, and 4 varieties of liverworts. A total of 86 species were listed for the first time as flora of Jeju Island. The list of species, with appropriate explanations, is provided below.

### 2.1. List of Taxa

**Figure 1:**
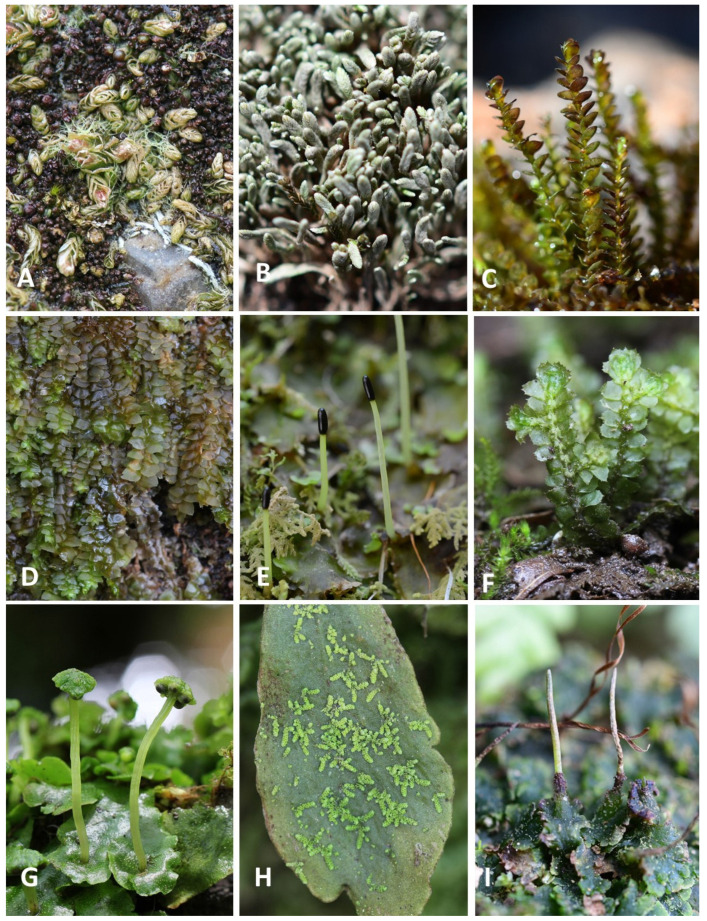
Some liverworts on Jeju Island. (**A**) *Cryptocoleopsis imbricata*; (**B**) *Gymnomitrion faurianum*; (**C**) *Solenostoma faurieanum*; (**D**) *Eocalypogeia quelpaertensis*; (**E**) *Makinoa crispata*; (**F**) *Porella japonica*; (**G**) *Wiesnerella denudata*; (**H**) *Cololejeunea subkodamae*; (**I**) *Folioceros fuciformis* (Photo by S.S. Choi 2010–2022).

The taxa are arranged alphabetically, and the nomenclature mostly follows Söderström et al. [11] with some updates from the recent literature. Each species is annotated by (1) altitudinal range, where the species was collected (in the case of a limited number of collection localities that were far from each other, the altitudes are given separated by commas), (2) field numbers of selected specimens examined (only one per locality is cited), (3) accompanying taxa (if there were any), (4) literature reference, if the species was treated in a previous record on the area, and the notes on some taxa (if any). There are 209 species, 9 subspecies and 4 varieties of liverworts and hornworts, of which 86 species that we found were new records for the island. Species newly recorded for Jeju Island are marked with asterisks (*), and the 27 species marked by the ° symbol represent the taxa reported in the literature but absent in our collected specimens. Some species’ habits are shown in Figure 1.

#### 2.1.1. Anthocerotae

*Anthoceros angustus Steph.—898 m—Moist shaded rocks near the stream valley—Selected specimens examined: *Choi 121016, 121017, 120320, 120321* (JNU).

*Anthoceros subtilis Steph.—898 m—Moist shaded rocks near the stream valley—Selected specimens examined: *Choi 121015* (JNU).

*Folioceros fuciformis (Mont.) D.C.Bhardwaj—172–864 m—Moist shaded rocks near the stream valley—Selected specimens examined: *Choi 8866, 8876, 111363, 111399, 111382* (JNU).

*Notothylas orbicularis (Schwein.) Sull.—300 m—Moist shaded soil near the trail in Gotjawal—Selected specimens examined: *Choi 110955* (JNU).

***Phaeoceros carolinianus*** (Michx.) Prosk.—32–898 m—Moist shaded rocks near the stream valley—Selected specimens examined: *Choi 7865, 8830, 8853, 8860, 111364, 111397, 111286, 120215, 121011, 121020* (JNU)—Accompanying species: *Scapania parvitexta*, *Calypogeia tosana*—Previously reported by Stephani [11] as *Anthoceros koreanus*, Song and Yamada [4].

*Phaeoceros laevis (L.) Prosk.—143 m—Moist shaded soil near the trail in Gotjawal—Selected specimens examined: *Choi 120027* (JNU).

*Megaceros flagellaris (Mitt.) Steph.—653 m—Moist shaded rocks in *Daphniphyllum macropodum* dominated forest in the stream valley—Selected specimens examined: *Choi 111485* (JNU).

#### 2.1.2. Hepaticae

***Acrolejeunea pusilla*** (Steph.) Grolle et Gradst.—329–1096 m a.s.l.—Shaded bark of tree near the stream valley—Selected specimens examined: *Choi 121079, 11082, 8002, 80045, 110974* (JNU)—Previously reported by Song and Yamada [10].

***Acrolejeunea sandvicensis*** (Gottsche) Steph.—1–1687 m a.s.l.—Shaded bark of tree and rocks—Selected specimens examined: *Choi 111207, 111500, 120709, 8852, 120004, 8063, 110907, 111002, 120171, 120073a, 120085, 120234, 120048, 120259* (JNU)—Previously reported by Hong [7] as *Brachiolejeunea sandvicensis*, Song and Yamada [10] as *Trocholejeunea sandvicensis*.

*Alobiellopsis parvifolia (Steph.) R.M. Schust.—461–470 m a.s.l.—Moist shaded soil near the stream valley—Selected specimens examined: *Choi 7604, 7608, 110973* (JNU).

*Aneura maxima (Schiffn.) Steph.—1230 m a.s.l.—Moist shaded soil near the stream valley—Selected specimens examined: *Choi 111423, 120753* (JNU).

*Aneura pinguis (L.) Dumort.—558 m a.s.l.—Moist shaded rocks near the stream valley—Selected specimens examined: *Choi 120112* (JNU).

*Anthelia juratzkana (Limpr.) Trevis.—1814 m a.s.l.—Shaded rocks near the top of Hallasan—Selected specimens examined: *Choi 120810 120812, 120824* (JNU).—Accompanying species: *Marsupella vermiformis*, *Dactyloradula brunnea*, *Gymnomitrion noguchianum*.

***Apopellia endiviifolia*** (Dicks.) Nebel et D.Quandt—898–1145 m a.s.l.—Shaded rocks near the stream valley—Selected specimens examined: *Choi 120729, 120302, 120307, 120308b, 120329* (JNU)—Previously reported by Hong [6] as *Pellia fabbroniana*, Song and Yamada [10] as *Pellia endiviifolia*.

***Asperifolia arguta*** (Nees & Mont.) A.V. Troitsky, Bakalin and Maltseva—271–640 m a.s.l.—Shaded wet soil near the trail—Selected specimens examined: *Choi 7605, 8064, 110817, 111040, 120424* (JNU)—Previously reported by Hattori et al. [5], Hong [6,7], Choe [8,9], Song and Yamada [10].

***Bazzania denudata*** (Lindenb. et Gottsche) Trevis.—350–1861 m a.s.l.—Shaded rocks and bark of tree (mainly Abies koreana)—Selected specimens examined: *Choi 111175, 7729, 120838, 120930, 120427, 120887* (JNU)—Accompanying species: *Scapania ampliata*, *Douinia plicata*, *Plicanthus birmensis*—Previously reported by Hattori et al. [5] as *Bazzania ovifolia*, Hong [6] as *Bazzania ovifolia*, Hong [7] as *Bazzania denudata* subsp. *ovifolia*, Choe [8,12] as *Bazzania ovifolia*, Song and Yamada [10].

*Bazzania japonica (Sande Lac.) Lindb.—172–476 m a.s.l.—Shaded rocks near the stream valley—Selected specimens examined: *Choi 111389, 111724, 111745* (JNU)

***Bazzania parabidentula*** Bakalin—1563–1916 m a.s.l.—Shaded rocks and bark of tree (mainly *Abies koreana*)—Selected specimens examined: *Choi 7743, 7750, 120439, 120817, 120830, 120914* (JNU)—Previously reported by Hong and Kim [13] as *Bazzania bidentula*, Hong [6,7] as *Bazzania bidentula*, Choe [8,9] as *Bazzania bidentula*, Song and Yamada [10] as *Bazzania bidentula*.

***Bazzania pompeana*** (Sande Lac.) Mitt.—311–690 m a.s.l.—Shaded rocks near the stream valley—Selected specimens examined: *Choi 7609, 7650, 8892, 8848, 111498, 110923, 120428, 121026* (JNU)—Accompanying species: *Pallavicinia subciliata*, *Calypogeia tosana*—Previously reported by Hattori et al. [5], Hong [6,7], Choe [9], Song and Yamada [10].

*Bazzania tricrenata (Wahlenb.) Lindb.—1687–1916 m a.s.l.—Shaded rocks humus near the stream valley—Selected specimens examined: *Choi 7752, 7754, 111198, 111199, 111240, 120708* (JNU).

*Bazzania tridens (Reinw., Blume et Nees) Trevis.—62 m a.s.l.—Shaded humus near the stream valley—Selected specimens examined: *Choi 120245, 120246* (JNU). 

***Blasia pusilla*** L.—172–898 m a.s.l.—Shaded rocks covered thin soil near the stream valley—Selected specimens examined: *Choi 111369, 111254, 111315, 7707, 120305* (JNU)—Previously reported by Hong [6], Choe [9].

***Blepharostoma epilithicum*** Vilnet et Bakalin—1687 m a.s.l.—Shaded humus near the stream valley—Selected specimens examined: *Choi 111189* (JNU).

***Blepharostoma minus*** Horik.—82–898 m a.s.l.—Shaded humus near the stream valley—Selected specimens examined: *Choi 8834, 111283, 120208, 120255* (JNU)—Previously reported by Horikawa [14], Hattori et al. [5], Hong [7], Choe [9], Song and Yamada [10].

*Calycularia laxa Lindb. et Arnell—1693–1916 m a.s.l.—Shaded rocks covered thin soil near the stream valley—Selected specimens examined: *Choi 7760, 7763, 120443* (JNU).

*Calypogeia japonica Steph.—350–1599 m a.s.l.—Shaded wet soil near the stream valley—Selected specimens examined: *Choi 8838, 8850, 111030, 111407, 111428, 111431, 111030, 120785* (JNU). 

°***Calypogeia orientalis*** Buczkowska et Bakalin—Previously reported by Hong [7] as *Calypogeia trichomanis*, Choe [8,9] as *Calypogeia trichomanis*, Song and Yamada [10] as *Calypogeia azurea*.

***Calypogeia tosana*** (Steph.) Steph.—469–999 m a.s.l.—Shaded humus and rock covered thin soil near the stream valley—Selected specimens examined: *Choi 7610, 7696, 8011, 120413, 120429, 120975* (JNU)—Previously reported by Hattori et al. [5], Hong [6,7], Song and Yamada [10].

°***Calypogeia yoshinagana*** Steph.—Previously reported by Hattori et al. [5] as *Calypogeia tosana* var. *yoshinagana*, Hong [6] as *Calypogeia tosana* var. *yoshinagana*.

*Cavicularia densa Steph.—766 m a.s.l.—Shaded wet rock covered thin soil near the stream valley—Selected specimens examined: *Choi 120467* (JNU)—Accompanying species: *Plagiochila ovalifolia*, *Plectocolea infusca*.

*Cephalozia bicuspidata (L.) Dumort.—1861 m a.s.l.—Shaded wet humus near the stream valley—Selected specimens examined: *Choi 120920* (JNU).

*Cephalozia lacinulata (J.B.Jack ex Gottsche et Rabenh.) Spruce.—1492 m a.s.l.—Decaying wood near the stream valley—Selected specimens examined: *Choi 111178* (JNU).

***Cephalozia otaruensis*** Steph.—72–1814 m a.s.l.—Shaded wet rock and soil near the stream valley—Selected specimens examined: *Choi 7614, 111754, 111215, 121063, 120823, 120872* (JNU)—Previously reported by Song and Yamada [10].

*Cephaloziella microphylla (Steph.) Douin—80–461 m a.s.l.—Shaded soil near the trail—Selected specimens examined: *Choi 7601, 120883* (JNU).

***Cephaloziella spinicaulis*** Douin—101–401 m a.s.l.—Shaded humus and decaying wood near the trail—Selected specimens examined: *Choi 111000, 120062, 121131* (JNU)—Previously reported by Hattori et al. [5] as *Cephaloziella echinata*, Song and Yamada [10].

*Cephaloziella spinigera (Lindb.) Jørg.—1230 m a.s.l.—Shaded humus and decaying wood near the trail—Selected specimens examined: *Choi 120749* (JNU).

***Cheilolejeunea nipponica*** (S. Hatt.) S. Hatt.—381–558 m a.s.l.—Shaded rocks near the trail—Selected specimens examined: *Choi 120106, 120178, 120195* (JNU)—Previously reported by Song and Yamada [10].

***Cheilolejeunea obtusifolia*** (Steph.) S. Hatt.—469–1096 m a.s.l.—Shaded wet cliffs near the trail—Selected specimens examined: *Choi 7637, 121072, 121080, 120143, 120493* (JNU)—Accompanying species: *Marsupella pseudofunkii*, *Herbertus aduncus*.

***Cheilolejeunea trapezia*** (Nees) Kachroo et R.M. Schust.—62 m a.s.l.—Shaded cliffs near the stream valley—Selected specimens examined: *Choi 120235* (JNU)—Previously reported by Hong [7] as *Cheilolejeunea imbricata*, Choe [9,12] as *Cheilolejeunea imbricata*, Song and Yamada [10].

***Chiastocaulon mayebarae*** (S. Hatt.) S.D.F. Patzak, M.A.M. Renner, Schäf.-Verw. & Heinrichs—1916 m a.s.l.—Shaded rocks near the stream valley *Choi 7764* (JNU)—Previously reported by Choe and Yamada [10].

***Chiloscyphus polyanthos*** (L.) Corda—32–1230 m a.s.l.—Opem and shaded submerged rocks near the stream valley—Selected specimens examined: *Choi 111440, 111409, 111436, 120752, 120024, 120001, 120211, 120212, 20495, 121021, 120330* (JNU)—Previously reported by Hattori et al. [5], Song and Yamada [10].

***Cladoradula auriculata*** (Steph.) M.A.M. Renner, Gradst., Ilk.-Borg. & F.R. Oliveira-da-Silva—476–1687 m a.s.l.—Shaded rocks near the stream valley—Selected specimens examined: *Choi 7714, 111742, 111200, 111221, 111222, 120990* (JNU)—Previously reported by Hong and Kim [13] as *Radula boryana*, Hattori et al. [5] as *Radula boryana*, Hong [6,7] as *Radula boryana*, Song and Yamada [10].

***Cololejeunea japonica*** (Schiffn.) Mizut.—101–680 m a.s.l.—Shaded bark of tree in evergreen broadleaved forest—Selected specimens examined: *Choi 7777, 110840, 121138, 121141, 120018, 120012, 120339, 111035, 110972, 120367, 120946* (JNU)—Previously reported by Song and Yamada [10].

°***Cololejeunea kodamae*** Kamim.—Previously reported by Hong and Kim [13], Hattori et al. [5], Hong [6,7], Choe [9], Song and Yamada [10].

*Cololejeunea longifolia (Mitt.) Benedix ex Mizut.—156–1096 m a.s.l.—Partly shaded rocks near the stream valley—Selected specimens examined: *Choi 8865, 8822, 8841, 120184, 120201,120086, 120088* (JNU)—Accompanying species: *Lejeuna japonica*.

***Cololejeunea macounii*** (Spruce) A. Evans—520–1916 m a.s.l.—Shaded rocks near the stream valley—Selected specimens examined: *Choi 7756, 7758, 111301, 111346, 120361b* (JNU)—Accompanying species: *Solenostoma rotundatum*.—Previously reported by Hong and Kim [13], Hattori et al. [5], Hong [6,7], Choe [9,12], Song and Yamada [10].

*Cololejeunea ornata A. Evans—766 m a.s.l.—Shaded rocks near the stream valley—Selected specimens examined: *Choi 120471* (JNU).

°***Cololejeunea planissima*** (Mitt.) Abeyw.—Previously reported by Horikawa 1939 as *Cololejeunea aoshimensis*, Hong [6,7] as *Cololejeunea aoshimensis*, Song and Yamada [10].

°***Cololejeunea raduliloba*** Steph.—Previously reported by Hong [6], Choe [9,12], Song and Yamada [10].

°***Cololejeunea shikokiana*** (Horik.) S. Hatt.—Previously reported by Choe [9,12], Song and Yamada [10].

***Cololejeunea subkodamae*** Mizut.—62–558 m a.s.l.—Shaded rocks near the stream valley—Selected specimens examined: *Choi 120960, 110924, 120063, 121028, 120123, 120229* (JNU)—Accompanying species: *Plagiochila sciophylla*, *Heteroscyphus planus*. 

***Conocephalum japonicum*** (Thunb.) Grolle—172–864 m a.s.l.—Shaded wet rocks near the stream valley—Selected specimens examined: *Choi 111366, 111377, 120479, 120498, 120973, 8868, 7706* (JNU)—Accompanying species: *Lophocolea minor*, *Plectocolea virgata*, *Porella vernicosa*—Previously reported by Hattori et al. [5] as *Conocephalum supradecompositum*, Choe [8] as *Conocephalum supradecompositum*, Song and Yamada [10].

***Conocephalum salebrosum*** Szweyk., Buczk. et Odrzyk.—172–766 m a.s.l.—Shaded wet rocks near the stream valley—Selected specimens examined: *Choi 7615, 8828, 111367, 110872, 121042, 120461, 120475* (JNU)—Previously reported by Hattori et al. [5] as *Conocephalum conicum*, Hong [6] as *Conocephalum conicum*, Song and Yamada [10] as *Conocephalum conicum*.

*Cryptocoleopsis imbricata Amakawa—1814 m a.s.l.—Shaded rocks near the top of Hallasan—Selected specimens examined: *Choi 201345, 201370* (JNU).

***Cylindrocolea recurvifolia*** (Steph.) Inoue—472–653 m a.s.l.—Shaded wet rocks near the stream valley—Selected specimens examined: *Choi 111323, 111327, 111329, 111447, 7669* (JNU)—Accompanying species: *Lepidozia vitrea*, *Syzygiella autumnalis*—Previously reported by Song and Yamada [10].

*Dactyloradula brunnea (Steph.) M.A.M. Renner & Gradst.—1814 m a.s.l.—Shaded rocks near the top of Hallasan mountain—Selected specimens examined: *Choi 120814a* (JNU).

*Diplophyllum albicans (L.) Dumort.—622–1916 m a.s.l.—Shaded humus and rocks near the stream valley—Selected specimens examined: *Choi 7761, 111345, 120918, 120922, 120931* (JNU).

°***Diplophyllum serrulatum*** (Müll. Frib.) Steph.—Previously reported by Hong [7], Choe [9], Song and Yamada [10]]

***Diplophyllum taxifolium*** (Wahlenb.) Dumort.—476–1747 m a.s.l.—Shaded humus and rocks near the stream valley—Selected specimens examined: *Choi 111767, 111302, 120764, 120802, 120706, 120508, 120979, 120304* (JNU)—Accompanying species: *Porella ulophylla*, *Radula constricta*—Previously reported by Amakawa and Hattori [15].

*Douinia plicata (Lindb.) Konstant. et Vilnet—1650 m a.s.l.—Shaded rocks in the stony field—Selected specimens examined: *Choi 111095* (JNU).

*Drepanolejeunea angustifolia (Mitt.) Grolle—1750 m a.s.l.—Shaded rocks in the stony field—Selected specimens examined: *Choi 111242* (JNU).

***Dumortiera hirsuta*** (Sw.) Nees—32–898 m a.s.l.—Shaded wet rocks near the stream valley and Gotjawal—Selected specimens examined: *Choi 7640, 8829, 120218, 121041, 120318, 111356* (JNU)—Accompanying species: *Heteroscyphus planus*, *Calypogeia tosana*, *Eocalypogeia querpaertensis*—Previously reported by Hattori et al. [5], Hong [6], Choe [8,9], Song and Yamada [10].

***Eocalypogeia quelpaertensis*** (S. Hatt. et Inoue) R.M. Schust.—77–898 m a.s.l.—Shaded wet rocks near the stream valley and Gotjawal—Selected specimens examined: *Choi 8844, 111384, 111390, 110826, 111484, 120207, 120364, 120879, 120308* (JNU)—Previously reported by Hattori et al. [5] as *Metacalypogeia quelpaertensis*, Hong [7] as *Metacalypogeia quelpaertensis*, Choe [8,9,12] as *Metacalypogeia quelpaertensis*, Song and Yamada [10].

*Fossombronia japonica Schiffn.—138–618 m a.s.l.—Shaded wet soil near the trail—Selected specimens examined: *Choi 8005, 8006, 8009, 8061, 8065, 8800* (JNU).

***Frullania appendiculata*** Steph.—401–1814 m a.s.l.—Partly open rocks and bark of tree—Selected specimens examined: *Choi 110828, 121094, 120816, 120825, 120140, 120087* (JNU)—Previously reported by Hattori et al. [5] as *Frullania tamarisci* subsp. *monilata*, Hong [6] as *Frullania tamarisci* subsp. *monilata*, Song and Yamada [10] as *Frullania tamarisci* subsp. *obscura*.

*Frullania crispiplicata Yuzawa et S. Hatt.—1230 m a.s.l.—Partly shaded bark of tree—Selected specimens examined: *Choi 120750* (JNU).

***Frullania davurica*** Hampe ex Gottsche, Lindenb. et Nees—401–766 m a.s.l.—Partly shaded bark of tree—Selected specimens examined: *Choi 120079, 120129, 120154, 120462* (JNU)—Previously reported by Hong [7], Song and Yamada [10].

°***Frullania densiloba*** Steph. ex A.Evans—Previously reported by Choe [9], Song and Yamada [10].

°***Frullania diversitexta*** Steph.—Previously reported by Choe [9], Song and Yamada [10].

°***Frullania ericoides*** (Nees) Mont.—Previously reported by Choe [9], Song and Yamada [10].

*Frullania fauriana Steph.—1230 m a.s.l.—Partly shaded bark of tree—Selected specimens examined: *Choi 120748* (JNU).

*Frullania fuscovirens Steph.—864 m a.s.l.—Partly shaded bark of tree—Selected specimens examined: *Choi 120998* (JNU).

***Frullania hamatiloba*** Steph.—766–898 m a.s.l.—Partly shaded bark of tree—Selected specimens examined: *Choi 120492, 121007, 120297, 120335* (JNU)—Previously reported by Hong [7], Song and Yamada [10].

***Frullania inflata*** Gottsche—97–195 m a.s.l.—Partly shaded bark of tree—Selected specimens examined: *Choi 121142, 120016, 120013, 120270, 120272, 120276, 120279* (JNU)—Previously reported by Song and Yamada [10].

°***Frullania kagoshimensis*** Steph.—Previously reported by Hong [7], Song and Yamada [10].

***Frullania muscicola*** Steph.—97–1230 m a.s.l.—Partly shaded bark of tree—Selected specimens examined: *Choi 7603, 110829, 110839, 111499, 121144, 120757, 120455, 120252, 120275* (JNU)—Previously reported by Hattori et al. [5], Hong [6,7], Choe [9] as *Frullania muscicola* var. *inuena*, Song and Yamada [10].

°***Frullania nepalensis*** (Spreng.) Lehm. et Lindenb.—Previously reported by Horikawa [16] as *Frullania nishiyamensis*, Song and Yamada [10].

***Frullania osumiensis*** (S. Hatt.) S. Hatt.—520 m a.s.l.—Partly shaded bark of tree—Selected specimens examined: *Choi 120362* (JNU)—Previously reported by Hong [7], Song and Yamada [10].

*Frullania pedicellata Steph.—992 m a.s.l.—Partly shaded bark of tree—Selected specimens examined: *Choi 8031* (JNU).

*Frullania polyptera Taylor—992–1916 m a.s.l.—Partly shaded bark of tree—Selected specimens examined: *Choi 7775, 8025, 8030, 8032* (JNU).

***Frullania schensiana*** C. Massal.—992 m a.s.l.—Partly shaded bark of tree—Selected specimens examined: *Choi 8021* (JNU)—Previously reported by Choe [9].

***Frullania taradakensis*** Steph.—401–864 m a.s.l.—Partly shaded bark of tree—Selected specimens examined: *Choi 120999, 120074b* (JNU)—Accompanying species: *Porella ulophylla*, *Lejeunea japonica*—Previously reported by Hong [7], Song and Yamada [10].

***Frullania usamiensis*** Steph.—1371 m a.s.l.—Partly shaded bark of tree—Selected specimens examined: *Choi 7712* (JNU)—Previously reported by Choe [9].

*Fuscocephaloziopsis lunulifolia (Dumort.) Váňa et L.Söderstr.—1371–1693 m a.s.l.—Shaded decaying wood near the stream valley—Selected specimens examined: *Choi 7722, 120447* (JNU).

*Geocalyx lancistipulus (Steph.) S. Hatt.—484–1687 m a.s.l. Shaded decaying wood near the stream valley—Selected specimens examined: *Choi 111280, 111192, 120157* (JNU).

*Gymnomitrion commutatum (Limpr.) Schiffn.—1750–1861 m a.s.l.—Partly shaded rocks near the top of mountain—Selected specimens examined: *Choi 111250, 120835, 120818, 120828, 120924, 120917a* (JNU)—Accompanying species: *Solenostoma pyriflorum*, *Diplophyllum albicans*.

*Gymnomitrion faurianum (Steph.) Horik.—1750–1916 m a.s.l.—Partly shaded rocks near the top of mountain—Selected specimens examined: *Choi 7765, 7759, 111241, 111245, 120704, 120827, 120894, 120907, 120937* (JNU).

*Gymnomitrion noguchianum S. Hatt.—1814 m a.s.l.—Shaded rocks near the top of mountain—Selected specimens examined: *Choi 120819, 120809, 120826* (JNU).

*Gymnomitrion parvitextum (Steph.) Mamontov, Konstant. et Potemkin—1861 m a.s.l.—Partly shaded rocks near the top of mountain—Selected specimens examined: *Choi 120917b* (JNU).

*Haplomitrium mnioides (Lindb.) R.M. Schust.—469 m a.s.l.—Shaded wet rocks near the stream valley and Gotjawal—Selected specimens examined: *Choi 7613* (JNU)—Accompanying species: *Lejeunea parva*, *Plectocolea infusca*.

***Herbertus aduncus*** (Dicks.) Gray—476–1916 m a.s.l.—Partly shaded rocks—Selected specimens examined: *Choi 7764, 111761, 111244, 111252, 120900, 120915, 120927, 120934* (JNU)—Previously reported by Hattori et al. [5] as *Herbertus hutchinsiae* subsp. *schusteri*, Hong [7] as *Herbertus hutchinsiae* subsp. *schusteri*, Song and Yamada [10].

*Herbertus buchii Juslén—1916 m a.s.l.—Partly shaded rocks near the top of mountain—Selected specimens examined: *Choi 7762* (JNU).

***Heteroscyphus argutus*** (Reinw., Blume et Nees) Schiffn.—50–149 m a.s.l.—Shaded wet rocks near the stream valley and Gotjawal—Selected specimens examined: *Choi 120710, 120037, 120874, 120047a* (JNU)—Accompanying species: *Lejeunea anisophylla*—Previously reported by Song and Yamada [10].

***Heteroscyphus coalitus*** (Hook.) Schiffn.—274–898 m a.s.l.—Shaded wet rocks near the stream valley and Gotjawal—Selected specimens examined: *Choi 7642, 8873, 8811, 110789, 120967, 120196, 121035, 121023, 120306* (JNU)—Accompanying species: *Acrolejeunea sandvicensis*, *Scapania integerrima*, *Lejeunea japonica*, *Plectocolea infusca*—Previously reported by Hattori et al. [5] as *Heteroscyphus bescherellei*, Hong [7] as *Heteroscyphus bescherellei*, Song and Yamada [10].

***Heteroscyphus planus*** (Mitt.) Schiffn.—62–618 m a.s.l.—Shaded wet rocks near the stream valley and Gotjawal—Selected specimens examined: *Choi 8805, 8882, 120023, 120230, 120239, 120875, 120249, 120284* (JNU)—Previously reported by Hong [6], Song and Yamada [10].

***Hygrobiella laxifolia*** (Hook.) Spruce—345–1230 m a.s.l.—Shaded wet rocks near the stream valley and Gotjawal—Selected specimens examined: *Choi 110851, 110854, 111267, 120734, 120481, 120490* (JNU)—Accompanying species: *Pellia neesiana*, *Plectocolea infusca*—Previously reported by Hattori et al. [12], Song and Yamada [10].

*Hygrobiella nishimurae N. Kitag.—350 m a.s.l.—Shaded wet rocks near the stream valley—Selected specimens examined: *Bakalin Kor-30–58-15* (VBGI).

***Jubula hutchinsiae* subsp. *japonica*** (Steph.) Horik. et Ando—523–558 m a.s.l.—Shaded wet rocks near the stream valley—Selected specimens examined: *Choi 8893, 121051, 12105b, 120161* (JNU)—Accompanying species: *Bazzania pompeana*—Previously reported by Song and Yamada [10].

*Jubula hutchinsiae subsp. *javanica* (Steph.) Verd.—311–381 m a.s.l.—Shaded wet rocks near the stream valley—Selected specimens examined: *Choi 110858, 110680, 110783, 110913* (JNU).

***Jungermannia atrovirens*** Dumort.—311–381 m a.s.l.—Shaded wet rocks near the stream valley—Selected specimens examined: *Choi 111350, 10847c, 120192, 120988* (JNU)—Accompanying species: *Marsupella apertifolia*, *Plectocolea ovalifolia*.

*Jungermannia pumila With.—558 m a.s.l.—Shaded wet rocks near the stream valley—Selected specimens examined: *Choi 9938b* (JNU).

***Kurzia makinoana*** (Steph.) Grolle—622–1814 m a.s.l.—Shaded humus near the stream valley—Selected specimens examined: *Choi 111319, 120831* (JNU)—Previously reported by Hattori et al. [5] as *Microlepidozia makinoana*, Hong [6,7] as *Microlepidozia makinoana*, Choe [8,9,12], Song and Yamada [10].

*Lejeunea anisophylla Mont.—32–622 m a.s.l.—Shaded wet rocks near the stream valley—Selected specimens examined: *Choi 111317, 120955, 120959, 120214, 120247, 12004b, 120876* (JNU)—Accompanying species: *Heteroscyphus argutus*.

*Lejeunea aquatica Horik.—32–558 m a.s.l.—Shaded submerged rocks in the stream valley—Selected specimens examined: *Choi 120021, 120025, 120213, 120219, 120036, 120168* (JNU).

***Lejeunea compacta*** (Steph.) Steph.—32–558 m a.s.l.—Shaded humus and rocks near the stream valley—Selected specimens examined: *Choi 111289, 111432, 111435, 120441* (JNU)—Previously reported by Horikawa [17] as *Euosmolejeunea auriculata*, Hattori et al. [5], Hong [6,7], Song and Yamada [10].

***Lejeunea discreta*** Lindenb.—401–558 m a.s.l.—Shaded humus and rocks near the stream valley—Selected specimens examined: *Choi 120060, 120061, 120067, 120102, 120132* (JNU)—Previously reported by Song and Yamada [10].

***Lejeunea flava*** (Sw.) Nees—381 m a.s.l.—Shaded humus and rocks near the stream valley—Selected specimens examined: *Choi 110819* (JNU)—Previously reported by Hong [7], Choe [9], Song and Yamada [10].

***Lejeunea japonica*** Mitt.—50–898 m a.s.l.—Shaded humus and rocks near the stream valley—Selected specimens examined: *Choi 8067, 8842, 111426, 120081, 120039, 12182, 120209, 120080* (JNU)—Accompanying species: *Lejeunea parva*—Previously reported by Hattori et al. [5], Hong [6,7], Song and Yamada [10].

*Lejeunea otiana S. Hatt.—50–898 m a.s.l.—Shaded humus and rocks near the stream valley—Selected specimens examined: *Choi 120005, 120019, 120353, 110977, 120129* (JNU).

***Lejeunea parva*** (S. Hatt.) Mizut.—81–653 m a.s.l.—Shaded humus and rocks near the stream valley—Selected specimens examined: *Choi 7612, 7636, 111337, 111441, 120957, 120059, 120141, 120153, 120884* (JNU)—Accompanying species: *Jubula japonica*—Previously reported by Hattori et al. [5] as *Lejeunea rotundistipula*, Hong [6,7] as *Lejeunea rotundistipula*, Song and Yamada [10].

°***Lejeunea planiloba*** A.Evans—Previously reported by Hong [7], Choe [9,12], Song and Yamada [10].

°***Lepidozia fauriana*** Steph.—Previously reported by Hong [7], Choe [9,12], Song and Yamada [10].

***Lepidozia reptans*** (L.) Dumort.—1499–1916 m a.s.l.—Shaded humus and decaying wood—Selected specimens examined: *Choi 7734, 7738, 7773, 111185b, 111204b, 120446, 120449b* (JNU)—Accompanying species: *Trichocoleopsis sacculata*, *Ptilidium pulcherrimum*, *Marsupella tubulosa*—Previously reported by Hong [7], Choe [8,9].

°***Lepidozia subtransversa*** Steph.—Previously reported by Stephani [18] as *Lepidozia coreana*, Song and Yamada [10].

***Lepidozia vitrea*** Steph.—156–653 m a.s.l.—Shaded humus and wet rock near the stream valley—Selected specimens examined: *Choi 7617, 7650, 7675, 8864, 110795, 111255, 111471, 111472, 120357, 121038, 121043, 120426* (JNU)—Previously reported by Hattori et al. [5], Hong [6,7], Choe [8,9], Song and Yamada [10].

***Liochlaena subulata*** (A. Evans) Schljakov—476–1563 m a.s.l.—Shaded humus and wet rock near the stream valley—Selected specimens examined: *Choi 7744, 11725* (JNU)—Previously reported by Hong [7] as *Jungermannia lanceolata* subsp. *stephanii*, Song and Yamada [10] as *Jungermannia subulata*.

***Lophocolea bidentata*** (L.) Dumort.—345–1563 m a.s.l.—Shaded humus and decaying wood near the stream valley—Selected specimens examined: *Choi 7739, 110847, 110850, 111702, 111011* (JNU)—Previously reported by Hattori et al. [5] as *Lophocolea cuspidata*, Hong [6,7] as *Lophocolea cuspidata*, Choe [9,12] as *Lophocolea cuspidata*, Song and Yamada [10].

***Lophocolea heterophylla*** (Schrad.) Dumort.—350–1563 m a.s.l.—Shaded humus and decaying wood near the stream valley—Selected specimens examined: *Choi 7740, 8801, 111009, 111021, 120127* (JNU)—Previously reported by Hattori et al. [5], Song and Yamada [10].

***Lophocolea horikawana*** S. Hatt.—1617–1916 m a.s.l.—Shaded humus and decaying wood near the stream valley—Selected specimens examined: *Choi 7746, 7748, 111216, 120799, 120903* (JNU)—Previously reported by Hong [6,7], Choe [9,12], Song and Yamada [10].

°***Lophocolea itoana*** Inoue—Previously reported by Choe [9].

***Lophocolea minor*** Nees—97–1687 m a.s.l.—Shaded decaying wood and wet rocks near the stream valley—Selected specimens examined: *Choi 8028, 8033, 110840b, 111732, 111205, 121061, 120131, 120144, 120285* (JNU)—Accompanying species: *Cololejeunea japonica*, *Metzgeria temperate*, *Plectocolea virgate*, *Porella vernicosa*, *Conocephalum japonicum*—Previously reported by Hong and Kim [13], Hattori et al. [5], Hong [7], Song and Yamada [10].

***Lophozia guttulata*** (Lindb. et Arnell) A. Evans—1563–1916 m a.s.l.—Shaded decaying wood near the stream valley—Selected specimens examined: *Choi 7741, 7749, 7769, 7772, 111237* (JNU)—Previously reported by Hattori et al. [5] as *Lophozia porphyroleuca*, Hong [6,7] as *Lophozia porphyroleuca*, Choe [8,9,12], Song and Yamada [10] as *Lophozia longiflora*.

*Lophoziopsis excisa (Dicks.) Konstant. et Vilnet—1747 m a.s.l.—Partly shaded rocks in the stony field—Selected specimens examined: *Choi 120701* (JNU).

*Lunularia cruciata (L.) Dumort. ex Lindb.—201 m a.s.l.—Open and shaded wet soil in greenhouse—Selected specimens examined: *Choi 110361b* (JNU).

***Makinoa crispata*** (Steph.) Miyake—653–864 m a.s.l.—Shaded wet rocks near the stream valley—Selected specimens examined: *Choi 111495, 120466, 120473, 120989* (JNU)—Previously reported by Hong [6], Choe [9], Song and Yamada [10].

***Marchantia emarginata* subsp. *tosana*** (Steph.) Bischl.—32–166 m a.s.l.—Shaded moisture soil near the roadside—Selected specimens examined: *Choi 8875, 8877, 8859, 8861, 120028, 120210* (JNU)—Previously reported by Choe [9,12] as *Marchantia tosana*, Song and Yamada [10].

*Marchantia paleacea subsp. *diptera* (Nees et Mont.) Inoue—172 m a.s.l.—Shaded moisture rocks near the stream valley—Selected specimens examined: *Choi 111362, 111379* (JNU).

*Marchantia paleacea subsp. *paleacea* Bertol.—32–142 m a.s.l.—Shaded moisture rocks near the stream valley—Selected specimens examined: *Choi 120217, 120970* (JNU).

***Marchantia polymorpha* subsp. *ruderalis*** Bischl. et Boissel.-Dub.—50–898 m a.s.l.—Shaded moisture soil—Selected specimens examined: *Choi 8827, 8874, 111029, 120199, 120035, 120044, 121009, 120319* (JNU).

*Marsupella apertifolia Steph.—640–1861 m a.s.l.—Shaded rocks near the stream valley—Selected specimens examined: *Choi 120904, 120419, 120847, 121107* (JNU).

*Marsupella koreana Bakalin et Fedosov—1492 m a.s.l.—Shaded rocks near the stream valley—Selected specimens examined: *Choi 111147* (JNU).

***Marsupella pseudofunckii*** S. Hatt.—520–1916 m a.s.l.—Shaded rocks near the stream valley—Selected specimens examined: *Choi 7757, 111223, 121064, 121077, 121078, 120703, 120358, 20417* (JNU)—Previously reported by Hattori et al. [5], Hong [7], Choe [9], Song and Yamada [10].

***Marsupella tubulosa*** Steph.—520–1916 m a.s.l.—Shaded rocks near the stream valley—Selected specimens examined: *Choi 8879, 8827, 7745, 120721, 120795* (JNU)—Previously reported by Hattori et al. [5], Hong [6,7] as *Marsupella emarginata* subsp. *tubulosa*, Song and Yamada [10] *Marsupella emarginata* subsp. *tubulosa*.

*Marsupella vermiformis (R.M. Schust.) Bakalin et Fedosov—1861 m a.s.l.—Shaded rocks near the stream valley—Selected specimens examined: *Choi 120911, 120897* (JNU, VBGI).

*Marsupella yakushimensis (Horik.) S. Hatt.—350–1687 m a.s.l.—Shaded rocks near the stream valley—Selected specimens examined: *Choi 111041, 111042, 111231, 111213, 111175, 111478, 111299* (JNU).

***Metacalypogeia cordifolia*** (Steph.) Inoue—476–1622 m a.s.l.—Shaded humus and decaying wood near the stream valley—Selected specimens examined: *Choi 7713, 7727, 7731, 111764, 111180, 111193, 121076, 120804* (JNU).

*Metasolenostoma ochotense Vilnet et Bakalin—992 m a.s.l.—Shaded wet soil near the stream valley—Selected specimens examined: *Choi 111730* (JNU).

°***Metzgeria furcata*** (L.) Corda—Previously reported by Choe [8,9,12] as *Metzgeria decipiens* and *Metzgeria fauriana*, Song and Yamada [10] as *Metzgeria decipiens*.

°***Metzgeria leptoneura*** Spruce—Previously reported by Hattori et al. [5] as *Metzgeria hamata*, Hong [6] as *Metzgeria hamata*, Choe [8,12] as *Metzgeria hamata*, Song and Yamada [10].

***Metzgeria lindbergii*** Schiffn.—97–898 m a.s.l.—Partly shaded bark of tree—Selected specimens examined: *Choi 8802, 110987, 120194, 120254, 120292, 120289* (JNU)—Previously reported by Hattori et al. [5], Song and Yamada [10].

***Metzgeria pubescens*** (Schrank) Raddi—271–898 m a.s.l.—Partly shaded humus and rocks near the stream valley—Selected specimens examined: *Choi 7725, 120437* (JNU)—Previously reported by Hattori et al. [5], Hong [6], Choe 1975, Song and Yamada [10].

***Metzgeria temperata*** Kuwah.—345–1916 m a.s.l.—Partly shaded bark of tree—Selected specimens examined: *Choi 7767, 8023, 8026, 110849, 111707, 121097, 120942, 120069* (JNU)—Previously reported by Song and Yamada [10].

***Microlejeunea punctiformis*** (Taylor) Steph.—401–558 m a.s.l.—Partly shaded bark of tree near the stream valley—Selected specimens examined: *Choi 120134* (JNU)—Previously reported by Choe [9,12] as *Lejeunea ulicina*, Song and Yamada [10] as *Lejeunea ulicina*.

***Myriocoleopsis minutissima*** (Sm.) R.L.Zhu, Y.Yu et Pócs—401–558 m a.s.l.—Partly shaded bark of tree near the Oreum—Selected specimens examined: *Choi 110830, 110831* (JNU)—Previously reported by Song and Yamada [10] as *Cololejeunea minutissima*.

***Nardia assamica*** (Mitt.) Amakawa—271–1610 m a.s.l.—Shaded wet soil near the stream valley—Selected specimens examined: *Choi 7708, 8066, 111482, 120762, 121109, 120793* (JNU)—Previously reported by Hattori et al. [5] as *Nardia sieboldii*, Song and Yamada [10].

*Pallavicinia subciliata (Austin) Steph—172–690 m a.s.l.—Shaded wet rocks near the stream valley and Oreum—Selected specimens examined: *Choi 7607, 8828, 111365, 111474, 121031, 120205* (JNU).

°***Pedinophyllum truncatum*** (Steph.) Inoue—Previously reported by Hong and Kim [13], Song and Yamada [10]. 

***Pellia neesiana*** (Gottsche) Limpr.—350–1614 m a.s.l.—Shaded wet rocks near the stream valley and Oreum—Selected specimens examined: *Choi 7709, 7774, 8880, 111257, 222310, 120738, 120444, 120991* (JNU)—Accompanying species: *Lepidozia vitrea*, *Lejeunea parva*—Previously reported by Hattori et al. [5], Hong [6], Choe [9], Song and Yamada [10].

*Plagiochila furcifolia Mitt.—149 m a.s.l.—Partly shaded bark of tree near the Oreum—Selected specimens examined: *Choi 120431* (JNU).

°***Plagiochila gracilis*** Lindenb. et Gottsche—Previously reported by Hattori et al. [5] as *Plagiochila firma* subsp. *rhizophora*, Hong [6] as *Plagiochila rhizophora*, Song and Yamada [10].

***Plagiochila hakkodensis*** Steph.—350–653 m a.s.l.—Shaded wet rocks near the stream valley and Oreum—Selected specimens examined: *Choi 111005, 111013, 111016, 111022, 111039, 111486b* (JNU)—Accompanying species: *Plagiochila porelloides*—Previously reported by Hattori et al. [5], Hong [6], Choe [9], Song and Yamada [10].

***Plagiochila ovalifolia*** Mitt.—258–1230 m a.s.l.—Shaded wet rocks near the stream valley and Oreum—Selected specimens examined: *Choi 7632, 8837, 110845, 110670, 111312, 111483, 121069, 121147, 120350, 120296* (JNU)—Accompanying species: *Marsupella tubulosa*, *Plectocolea infusca*, *Lejeuena japonica*—Previously reported by Hattori et al. [5], Hong [6] as *Plagiochila ovalifolia* var. *miyoshiana*, Hong [7] as *Plagiochila asplenioides* subsp. *ovalifolia* and *Plagiochila quelpaertensis*, Choe [9,12] as *Plagiochila quelpaertensis*, Song and Yamada [10].

***Plagiochila porelloides*** (Torr. ex Nees) Lindenb.—370–640 m a.s.l.—Shaded wet rocks near the stream valley and Oreum—Selected specimens examined: *Choi 110861, 111486, 120428* (JNU)—Accompanying species: *Bazzania pompeana*, *Pallavicinia subciliata*—Previously reported by Hong and Kim [13] as *Plagiochila satoi*, Hattori et al. [5] as *Plagiochila satoi*, Hong [6,7] as *Plagiochila satoi*, Song and Yamada [10].

***Plagiochila sciophila*** Nees—62–618 m a.s.l.—Shaded wet rocks near the stream valley and Oreum—Selected specimens examined: *Choi 7688, 8804, 8810, 110995, 111019, 120071, 120117, 120257, 120108* (JNU)—Previously reported by Hattori et al. [5] as *Plagiochila japonica*, Hong [6,7] as *Plagiochila japonica*, Song and Yamada [10].

°***Plagiochila semidecurrens*** (Lehm. et Lindenb.) Lindenb.—Previously reported by Hattori et al. [5] as *Plagiochila semidecurrens* var. *grossidens*, Hong [6,7], Song and Yamada [10].

*Plagiochila shangaica Steph.—63 m a.s.l.—Shaded rocks near the stream valley—Selected specimens examined: *Choi 120045, 120046* (JNU).

*Plagiochila trabeculata Steph.—345 m a.s.l.—Shaded rocks near the stream valley—Selected specimens examined: *Choi 110859* (JNU).

*Plectocolea comata (Nees) S. Hatt.—172 m a.s.l.—Shaded wet rocks near the stream valley—Selected specimens examined: *Choi 8061, 111391* (JNU).

*Plectocolea erecta Amakawa—1687 m a.s.l.—Shaded wet rocks near the stream valley—Selected specimens examined: *Choi 110379* (JNU).

*Plectocolea granulata (Steph.) Bakalin—992 m a.s.l.—Shaded wet soil near the stream valley—Selected specimens examined: *Choi 8019* (JNU).

*Plectocolea grossitexta (Steph.) S. Hatt.—992 m a.s.l.—Shaded wet soil near the stream valley—Selected specimens examined: *Choi 8057* (JNU).

***Plectocolea infusca* var. *infusca*** Mitt.—461–1230 m a.s.l.—Shaded wet rocks near the stream valley—Selected specimens examined: *Choi 8061, 8823, 111429, 111734* (JNU)—Previously reported by Hong [7] as *Jungermannia infusca*, Song and Yamada [10] as *Jungermannia infusca*.

***Plectocolea infusca* var. *recondita*** Bakalin—1230 m a.s.l.—Shaded wet rocks near the stream valley—Selected specimens examined: *Choi 111419* (JNU)—Previously reported by Hattori et al. [5] as *Jungermannia infusca* var. *ovicalyx*, Choe [9] as *Jungermannia infusca* var. *ovicalyx*, Song and Yamada [10] as *Jungermannia infusca* var. *ovicalyx*.

*Plectocolea kurilensis (Bakalin) Bakalin et Vilnet—156–1230 m a.s.l.—Shaded wet soil near the stream valley—Selected specimens examined: *Choi 8824a, 111448, 111383, 111259, 120737* (JNU).

°***Plectocolea ovalifolia*** (Amakawa) Bakalin et Vilnet—Previously reported by Hattori et al. [5] as *Jungermannia infusca* var. *ovalifolia*, Hong [6,7] as *Jungermannia infusca* var. *ovalifolia*, Choe [9] as *Jungermannia infusca* var. *ovalifolia*, Song and Yamada [10] as *Jungermannia infusca* var. *ovalifolia*.

***Plectocolea radicellosa*** (Mitt.) Mitt.—172–898 m a.s.l.—Shaded wet rocks near the stream valley—Selected specimens examined: *Choi 7627, 8824, 112265, 120324* (JNU)—Previously reported by Choe [9,12] as *Jungermannia radicellosa*.

*Plectocolea rosulans (Steph.) S. Hatt.—558–1916 m a.s.l.—Shaded wet rocks near the stream valley—Selected specimens examined: *Choi 7761, 7620, 8821, 8895, 111455* (JNU).

*Plectocolea truncata (Nees) Herzog—156–271 m a.s.l.—Shaded wet rocks near the stream valley—Selected specimens examined: *Choi 8059, 8878* (JNU).

***Plectocolea virgata*** Mitt.—156–800 m a.s.l.—Shaded wet rocks near the stream valley—Selected specimens examined: *Choi 7784b, 111260* (JNU)—Previously reported by Hattori et al. [5] as *Jungermannia virgata*, Hong [6], Hong [7] as *Jungermannia virgata*, Choe [9] as *Jungermannia virgata*, Song and Yamada [10] as *Jungermannia virgata*.

*Plicanthus birmensis (Steph.) R.M. Schust.—350–541 m a.s.l.—Open and Shaded dried rocks near the stream valley—Selected specimens examined: *Choi 111295, 111294, 111022b* (JNU).

***Porella caespitans* var. *cordifolia*** (Steph.) S. Hatt. ex T.Katag. et T.Yamag.—370–1371 m a.s.l.—Shaded dried rocks near the stream valley—Selected specimens examined: *Choi 7712, 8803, 120423, 111868, 120869* (JNU)—Previously reported by Hong et al. [19] as *Porella setigera*, Hattori et al. [5] as *Porella setigera*, Hong [6,7] as *Porella setigera*, Song and Yamada [10].

°***Porella densifolia*** (Steph.) S. Hatt.—Previously reported by Song and Yamada [10] as *Porella densifolia* var. *fallax*.

***Porella fauriei*** (Steph.) S. Hatt.—350–1499 m a.s.l.—Shaded dried rocks near the stream valley—Selected specimens examined: *Choi 7717, 7726, 111024* (JNU)—Previously reported by Hong et al. [19] as *Porella vernicosa* subsp. *fauriei*, Hattori et al. [5], Hong [6,7], Song and Yamada [10].

***Porella grandiloba*** Lindb.—350–618 m a.s.l.—Shaded dried rocks near the stream valley—Selected specimens examined: *Choi 8812, 8817, 110994* (JNU)—Previously reported by Hong et al. [19], Hattori et al. [5], Hong [6,7], Song and Yamada [10].

***Porella japonica*** (Sande Lac.) Mitt.—273–640 m a.s.l.—Shaded rocks near the Gotjawal—Selected specimens examined: *Choi 120103, 120104 120138, 110931* (JNU)—Accompanying species: *Radula obtusiloba*, *Plectocolea erecta*, *Pellia neesiana*—Previously reported by Hong et al. [19], Hattori et al. [5], Hong [6,7], Song and Yamada [10].

***Porella oblongifolia*** S. Hatt.—370 m a.s.l.—Shaded rocks near the Gotjawal—Selected specimens examined: *Choi 110880* (JNU)—Previously reported by Hong [7], Choe [9], Song and Yamada [10].

***Porella ulophylla*** (Steph.) S. Hatt.—97–1693 m a.s.l.—Shaded rocks and bark of tree—Selected specimens examined: *Choi 7708, 8068, 111497, 121140, 120278, 120491* (JNU)—Previously reported by Hong and Kim [13], Hong et al. 1961, Hattori et al. [5], Hong [6,7], Song and Yamada [10] as *Macvicaria ulophylla*.

***Porella vernicosa*** Lindb.—97–1916 m a.s.l.—Shaded rocks and bark of tree—Selected specimens examined: *Choi 7711, 7776, 110976, 111038, 120456, 120971, 120120* (JNU)—Accompanying species: *Pseudolophozia sudetica*, *Plectocolea infusca*, *Radula japonica*, *Blepharostoma minus*, *Marsupella tubulosa*—Previously reported by Hong et al. [19], Hattori et al. [5], Hong [6,7], Song and Yamada [10].

***Protosolenostoma fusiforme*** (Steph.) Vilnet et Bakalin—329–1916 m a.s.l.—Shaded wet rocks near the stream valley—Selected specimens examined: *Choi 7768, 8001, 8035, 111287, 111482, 111411, 120847, 121070* (JNU)—Previously reported by Stephani [20] as *Solenostoma koreanum*; Hong [7] as *Jungermannia koreana*, Choe [9,12] as *Jungermannia fusiformis*, Song and Yamada [10] as *Jungermannia fusiformis*.

*Pseudolophozia sudetica (Nees ex Huebener) Konstant. et Vilnet—1750 m a.s.l.—Shaded wet rocks near the stream valley—Selected specimens examined: *Choi 111032b* (JNU).

***Ptilidium pulcherrimum*** (Weber) Vain.—350–1916 m a.s.l.—Partly shaded bark of tree—Selected specimens examined: *Choi 7718, 7720, 111239, 121117, 111772, 120935* (JNU)—Previously reported by Hong and Kim [13], Hattori et al. 1962, Hong [6,7], Choe [8,9,12], Song and Yamada [10].

°***Radula cavifolia*** Hampe ex Gottsche—Previously reported by Hong [7], Choe [9], Song and Yamada [10].

***Radula constricta*** Steph.—476–1693 m a.s.l.—Partly shaded bark of tree near the stream valley—Selected specimens examined: *Choi 121005* (JNU)—Previously reported by Hattori et al. [5], Hong [6], Song and Yamada [10].

***Radula japonica*** Gottsche—62–766 m a.s.l.—Partly shaded rocks near the stream valley—Selected specimens examined: *Choi 8069, 8813, 120227, 120459, 120183* (JNU)—Previously reported by Hong [6,7], Song and Yamada [10].

***Radula kojana*** Steph.—142–653 m a.s.l.—Shaded wet rocks near the stream valley—Selected specimens examined: *Choi 110677, 111325, 111487, 111491, 120956, 7677, 8888* (JNU)—Previously reported by Hattori et al. [5], Hong [6,7], Choe [9,12], Song and Yamada [10].

***Radula obtusiloba*** Steph.—258–766 m a.s.l.—Shaded wet rocks near the stream valley—Selected specimens examined: *Choi 120507, 111010, 110989, 110846, 110852, 110867, 110980* (JNU)—Previously reported by Hong [6], Song and Yamada [10].

*Radula oyamensis Steph.—381–548 m a.s.l.—Partly shaded bark of tree near the Gotjawal—Selected specimens examined: *Choi 110820, 121054* (JNU).

***Radula tokiensis*** Steph.—180 m a.s.l.—Shaded wet rocks near the stream valley—Selected specimens examined: *Choi 121124* (JNU)—Previously reported by Hong [7], Song and Yamada [10].

***Reboulia hemisphaerica* subsp. *hemisphaerica*** (L.) Raddi—32–898 m a.s.l.—Shaded rocks covered thin soil near the stream valley—Selected specimens examined: *Choi 120327, 120494, 120221, 7681, 7695, 111371* (JNU)—Previously reported by Horikawa [17].

***Reboulia hemisphaerica* subsp. *orientalis*** R.M. Schust.—156–618 m a.s.l.—Shaded rocks covered thin soil near the stream valley—Selected specimens examined: *Choi 8806, 8835, 8889, 8871* (JNU)—Previously reported by Song and Yamada [10].

°***Riccardia chamedryfolia*** (With.) Grolle—Previously reported by Choe [9], Song and Yamada [10].

*Riccardia glauca Furuki—469–489 m a.s.l.—Shaded wet rocks near the stream valley *Choi 7631, 7647, 7676, 7680* (JNU).

*Riccardia planiflora (Steph.) S. Hatt.—864 m a.s.l.—Shaded wet rocks near the stream valley—Selected specimens examined: *Choi 121000* (JNU).

*Riccia beyrichiana Hampe—92 m a.s.l.—Partly shaded soil near the road—Selected specimens examined: *Choi 201063* (JNU).

*Riccia bifurca Hoffm.—92 m a.s.l.—Partly shaded soil near the road—Selected specimens examined: *Choi 201065b* (JNU).

*Riccia fluitans L.—138 m a.s.l.—Submerged soil in the small pond—Selected specimens examined: *Choi 8009* (JNU).

***Riccia glauca*** L.—172–271 m a.s.l.—Partly shaded soil near the road—Selected specimens examined: *Choi 8060, 8061* (JNU)—Previously reported by Choe [9], Song and Yamada [10].

*Riccia huebeneriana Lindenb.—155–271 m a.s.l.—Partly shaded wet soil near the road—Selected specimens examined: *Choi 8007, 8008, 8062* (JNU).

*Ricciocarpos natans (L.) Corda—69 m a.s.l.—Submerged soil in the small pond—Selected specimens examined: *Choi 5243* (JNU).

***Scapania ampliata*** Steph.—476–1861 m a.s.l.—Partly shaded hnumus and rocks—Selected specimens examined: *Choi 120702, 120820, 111703, 111740, 120928* (JNU)—Previously reported by Hong and Kim [13], Hattori et al. [5], Hong [6,7], Choe [9], Song and Yamada [10].

***Scapania ciliata*** Sande Lac.—476–1861 m a.s.l.—Partly shaded humus and rocks—Selected specimens examined: *Choi 120973b, 120974, 111708, 111728, 111493, 121074* (JNU)—Previously reported by Hattori et al. [5] as *Scapania spinosa*, Hong [6,7] as *Scapania spinosa*, Choe [9], Song and Yamada [10].

°***Scapania curta*** (Mart.) Dumort.—Previously reported by Hattori et al. [5], Hong [6], Song and Yamada [10].

***Scapania integerrima*** Steph.—476–1861 m a.s.l.—Partly shaded wet rocks near the stream valley—Selected specimens examined: *Choi 120074c, 120360, 120264, 110800, 111333* (JNU)—Previously reported by Hong [7] as *Scapania stephanii*, Song and Yamada [10] as *Scapania ligulata*.

***Scapania irrigua*** (Nees) Nees—1650–1916 m a.s.l.—Partly shaded wet rocks near the stream valley—Selected specimens examined: *Choi 7736, 111067, 120905, 120891* (JNU)—Previously reported by Song and Yamada [10].

*Scapania parvidens Steph.—520–1861 m a.s.l.—Partly shaded wet rocks near the stream valley—Selected specimens examined: *Choi 120365b, 120505, 120499, 120480, 120476, 120912* (JNU).

*Scapania parvitexta Steph.—351–1861 m a.s.l.—Partly shaded wet rocks near the stream valley—Selected specimens examined: *Choi 110674, 111705, 111304, 120790, 120314* (JNU).

***Scapania undulata*** (L.) Dumort.—351–1861 m a.s.l.—Partly shaded wet rocks near the stream valley—Selected specimens examined: *Choi 120720, 120724, 120779, 120794, 120846* (JNU)—Previously reported by Song and Yamada [10].

***Schistochilopsis cornuta*** (Steph.) Konstant.—1916 m a.s.l.—Partly shaded decaying wood—Selected specimens examined: *Choi 7769* (JNU)—Previously reported by Hattori et al. [5] as *Lophozia cornuta*, Hong [6,7] as *Lophozia cornuta*, Choe [8,9,12] as *Lophozia cornuta*, Song and Yamada [10] as *Lophozia cornuta*.

*Solenostoma bilobum (Amakawa) Potemkin et Nyushko—600–800 m a.s.l.—Shaded wet rocks near the stream valley—Selected specimens examined: *Choi 110919* (JNU), *Bakalin Kor-29–27-15* (VBGI).

***Solenostoma faurieanum*** (Beauverd) R.M. Schust.—1595–1916 m a.s.l.—Shaded wet rocks near the stream valley—Selected specimens examined: *Choi 7768, 120778, 120806* (JNU)—Previously reported by Stephani [20] as *Jungermannia decurrens*, Hong [6,7] as *Jungermannia fauriana*, Choe [9,12] as *Jungermannia fauriana*, Song and Yamada [10] as *Jungermannia fauriana*.

*Solenostoma minutissimum (Amakawa) Bakalin—541–1230 m a.s.l.—Shaded wet rocks near the stream valley—Selected specimens examined: *Choi 111307, 120745* (JNU).

*Solenostoma purpuratum (Mitt.) Steph. var. ***koponenii*** Bakalin et Li Wei—766 m a.s.l.—Shaded wet rocks near the stream valley—Selected specimens examined: *Choi 120375* (JNU).

***Solenostoma pyriflorum*** Steph.—600–800 m a.s.l.—Shaded wet rocks near the stream valley—Selected specimens examined: Bakalin Kor-29–66-15 (VBGI)—Previously reported by Hattori et al. [5] as *Jungermannia pyriflora*, Choe [9] as *Jungermannia pyriflora*, Song and Yamada [10] as *Jungermannia pyriflora*.

***Solenostoma rotundatum*** Amakawa—172 m a.s.l.—Shaded wet rocks near the stream valley—Selected specimens examined: *Choi 111381* (JNU)—Previously reported by Hong [7] as *Jungermannia harana*, Song and Yamada [10] as *Jungermannia rotundata*.

*Solenostoma sunii Bakalin et Vilnet—1230 m a.s.l.—Shaded wet rocks near the stream valley—Selected specimens examined: *Choi 111425* (JNU).

*Spruceanthus kiushianus (Horik.) X.Q.Shi, R.L.Zhu et Gradst.—195 m a.s.l.—Partly shaded rocks near the stream valley—Selected specimens examined: *Choi 120008* (JNU).

***Syzygiella autumnalis*** (DC.) K.Feldberg, Váňa, Hentschel et Heinrichs—273–1693 m a.s.l.—Shaded humus near the stream valley—Selected specimens examined: *Choi 120128, 111323, 111449, 111771, 111729* (JNU)—Previously reported by Hong and Kim [13] as *Jamesoniella autumnalis*, Hattori et al. [5] as *Jamesoniella autumnalis*, Song and Yamada [10] as *Jamesoniella autumnalis*.

*Syzygiella nipponica (S. Hatt.) K.Feldberg, Váňa, Hentschel et Heinrichs—1096–1661 m a.s.l.—Shaded humus near the stream valley—Selected specimens examined: *Choi 121062, 121118* (JNU).

***Trichocolea tomentella*** (Ehrh.) Dumort.—1670 m a.s.l.—Shaded wet humus near the stream valley—Selected specimens examined: *Choi 22000*—Previously reported by Hong [7], Choe [8,9,12].

***Trichocoleopsis sacculata*** (Mitt.) S.Okamura—476–1861 m a.s.l.—Shaded wet rocks and humus near the stream valley—Selected specimens examined: *Choi 120509, 120933, 1211132, 111191, 111188, 111710* (JNU)—Previously reported by Hattori et al. [12], Hong [7], Choe [8,9], Song and Yamada [10].

*Tritomaria exsecta (Schmidel) Schiffn. ex Loeske—1861–1916 m a.s.l.—Partly shaded decaying wood—Selected specimens examined: *Choi 7749, 7769, 120901* (JNU).

***Wiesnerella denudata*** (Mitt.) Steph.—172–766 m a.s.l.—Shaded wet rocks near the stream valley and Gotjawal—Selected specimens examined: *Choi 8833* (JNU)—Previously reported by Hong [6], Song and Yamada [10].

### 2.2. Phytogeographic Speculations

**Figure 2:**
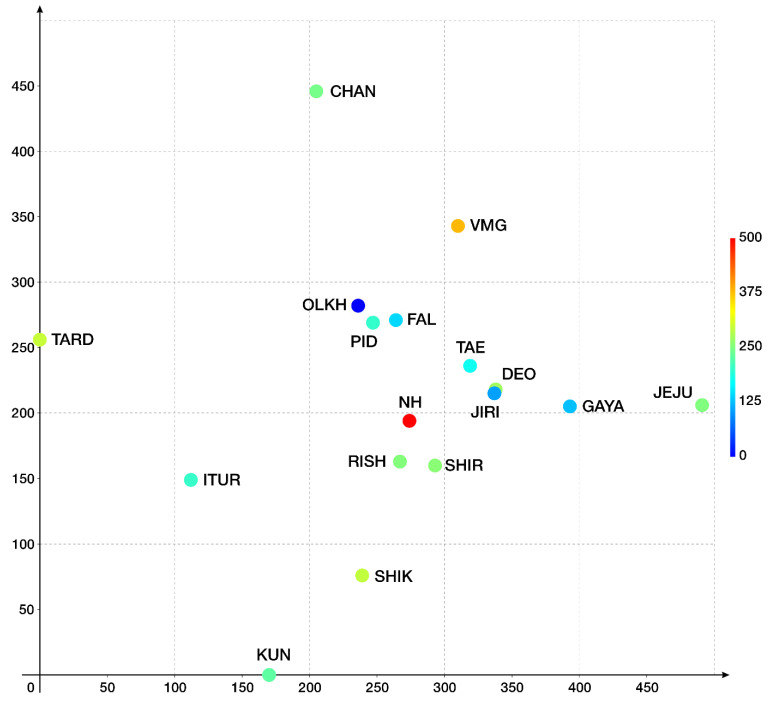
Comparison of the flora distribution in the DCA bubble chart (the third axis is the color gradient from deep blue to deep red).

The diagram obtained by DCA is shown in Figure 2. As seen from the diagram presented in Figure 2, some locality concentrations of the central floras were found in the northern extremity of the suboceanic sector of the East Asian floristic region: the area from the southern Sikhote-Alin to the central part of the Republic of Korea was reflected in the central part of the diagram. Two distinct floras were identified. The first was the flora of the Tardoki-Yani Range, which is situated beyond the northern limit of the East Asian region, although quite close to it. Second is the flora of the studied area (Jeju Island), where the number of conditionally “southern” species is the largest. In general, the abscissa axis is inversely proportional to the decrease in the latitude of the flora (arranged from the north to the south), while the ordinate axis may generally correspond to the gradient of oceanicity/continentality in climate (from insular floras at the bottom to the floras of the East Asian mainland). Table S1 also contains accompanying materials showing the distances in conventional units between the floras included in the analysis, including (1) data on distances in conventional units (in accordance with the distance between points in a three-dimensional grid) and (2) distances as a percentage of the average across the matrix calculated from the diagram.

The floras of the middle part of the diagram are most closely related to each other, and the distances between them vary within 74–81% of the average. The most closely situated floras of Pidan, Falaza, Deokgyu, Taebaeksan, Shirakami, and Rishiri (according to Table 1) show the closest relationships. The studied flora of Jeju Island is associated with other floras included in the analysis and, in general, is lower than the average distance along the matrix (the average distance between the Jeju Island flora and the other local floras is 117% of the average distance in the matrix). The floras most closely situated to the Jeju Island flora are the floras of the central part of the Republic of Korea: Deokgyu and Gayasan (68 and 69% of the average distance along the matrix, respectively). Other South Korean floras, although close to that of Jeju Island, are not so closely related (Jirisan—93%, Taebaeksan—83%). Of the non-Korean floras, the Shirakami (89%) and Rishiri (100%) floras show the closest relationship to that of Jeju Island. In general, the data obtained show a fairly high distance between the Jeju Island liverwort flora and most other floras included in the analysis.

## 3. Discussion

### 3.1. Vertical Distribution by Main Vegetation Zone

A crater (alt. 1800–1900 m) wall created by a volcanic eruption is located at the top of Hallasan Mountain (alt. 1950 m) on Jeju Island. The main habitat here consists of rock formations with no vegetation and is partly covered by *Abies koreana* stands. Additionally, *Diapensia lapponica* var. *obovata* and *Vaccinium uliginosum*, which are evaluated as a remnant plant of the ice age, grow here. *Anthelia juratzkana* is an arctic–alpine species that is regarded as a relic species on Jeju Island. In this vegetation zone, 44 taxa were collected.

Below the top area (alt. 1400–1800 m), a coniferous forest zone is distributed, and boreal species such as *Schistochilopsis cornuta*, *Tritomaria exsecta*, *Pseudolophozia sudetica*, *Lophoziopsis excisa*, and *Douinia plicata* are typical here. In this vegetation zone, 60 taxa were collected.

In the deciduous broadleaved forest area (750–1400 m), temperate–boreal species such as *Scapania ampliata*, *Scapania irrigua*, *Trichocolea tomentella*, *Lophozia guttulata*, and *Bazzania tricrenata* are distributed. In this vegetation zone, 98 taxa were collected.

Large and small Gotjawal and Oreums are distributed in the middle to low mountainous areas (alt. 200–750 m) of Jeju Island. In this area, evergreen broadleaved and deciduous broadleaved forests are mixed, and subtropical and temperate species such as *Haplomitrium mnioides*, *Marsupella yakushimensis*, *Porella grandiloba*, *Frullania davurica*, *Makinoa crispata*, *Cavicularia densa*, and *Cylindrocolea recurvifolia* are distributed. In this vegetation zone, 124 taxa were collected.

In the lowlands (alt. 0–200 m) of Jeju Island, warm–temperate evergreen broadleaved forests are distributed. In particular, in Seogwipo city, the southern part of Jeju Island, Lejeuneaceae species, such as *Lejeunea discreta*, *L. anisophylla, L. aquatica*, *L. otiana*, *Cheilolejeunea trapezia*, *Spruceanthus kiushianus*, and *Cheilolejeunea nipponica,* are distributed in tropical and subtropical regions. In this vegetation zone, 64 taxa were collected.

### 3.2. Geographical Specificity

*Marsupella vermiformis*, described in Papua New Guinea, where it was collected at elevations exceeding 4000 m a.s.l., may indicate peculiar cold alpine environments. It was found here on the shady and dry rocky surface of the northwest wall of Hallasan Mountain, and there is no explanation other than that it grows in a volcanic zone, similar to that of Papua New Guinea. *Cryptocoleopsis imbricata* grows on basalt rocks in the crater of Hallasan Mountain and ranges from Kamchatka to Japan. *Gymnomitrion noguchianum* and *Anthoceros subtilis* are not distributed in mainland Korea, China, and the Russian Far East but rather only in some areas of Japan and Jeju Island. *Marsupella koreana*, described as a new species [21], is distributed only on the mainland of Korea and Jeju Island.

### 3.3. Phytogeographic Speculations

Jeju Island is located in the warm temperate zone of the East Asian region. However, due to the regional features of the climate, the peculiar geographical position, and the mountainous relief, there is a mixture of plants from different vegetation zones. Among the liverwort taxa known in the studied area, there are both subtropical and even predominantly tropical species, as well as arctomontane taxa. Apparently, here, one can observe the shortest topographic distances between the species of both groups, for example, between the localities of *Cololejeunea planissima* and *Anthelia juratzkana*, with a distance of only 17.8 km by a direct line.

The closest relationships observed on the basis of the conducted DCA for the Jeju Island liverwort flora are with the mountain floras of the central part of the Republic of Korea, which, in general, is understandable in terms of geographical proximity. At the same time, the relationship with the floras of other regions is much less pronounced (with the exception of two floras of northern Japan). The Jeju Island flora occupies a position among the floras of the peninsular part of warm temperate Asia; however, it is not closely related to them, either. Being at the southernmost tip of Korea, the characteristics of the flora seem to be intermediate between the floras of the southern tip of the Korean Peninsula and other warm-temperate floras located further south in Japan and China. The distinct relationships are difficult to evaluate due to limited data on southwardly adjacent local floras of similar size.

## 4. Materials and Methods

### 4.1. Study Site

Jeju Island is located about 90 km south of the Korean Peninsula, at the southwest entrance of the Korea Strait. Jeju Island is the largest island in the Korean Peninsula, with an area of about 1828 km^2^ excluding attached islands. Jeju Island is a volcanic island formed by volcanic activity between approximately 1.8 million and 1000 years ago, roughly from the end of the tertiary period to the beginning of the 4th period of the Cenozoic Era [1,22]. The advance and retreat of the sea during the glacial and interglacial periods brought Jeju Island and the Korean Peninsula and other surrounding areas into contact with land and then isolated them. The depth of water between Jeju Island and the Korean Peninsula is around 150 m, and that fact can be known. After the glacial period, Jeju Island became a volcanic island separated from the Korean peninsula, and indigenous plants and remnants of the post-glacial period grow there [23].

Hallasan Mountain is located in the center of Jeju Island, with an elevation of 1950 m above sea level, and is the highest mountain in the Republic of Korea. Jeju Island, created by volcanic activity, boasts a diverse and unique volcanic landscape. There are approximately 360 large and small oreums (small volcanic hills on Jeju Island) spread out above the ground, and 160 lava caves are scattered all over the island below the ground [22]. 

The climate of Jeju Island changes from subtropical at low elevations to warm temperate, temperate, and, finally, subarctic on Mt. Halla in the center of the island. In addition, the ecosystem composition and species of the entire island are very diverse due to geographical and regional factors, and it possesses diverse topographical elements such as valleys, oreums, and Gotjawal (the local Jeju Island word for various types of vegetation, such as forests and thickets intertwined with a rock formation) formed by volcanic activity.

Jeju Island belongs to the East Asian Floristic Region of the Holarctic Kingdom [24]. Moreover, as a result of its unique geographic and regional location, Jeju Island is also a distribution boundary for the elements of the Indo-Malaysian subkingdom, as well as the East Siberian floristic region [25,26]. Therefore, the plants of Jeju Island are plants that reflect various factors, such as those related to the flora that descended from the continent, the flora distributed throughout China, Jeju Island, and Japan, the plants of tropical and subtropical origin, and the flora that differentiated on the islands of Jeju, Taiwan, and Japan. It can be seen that many species are widely distributed beyond the area that was shown based on the vascular plant study by Im [23].

The plants of Jeju Island growing in its various climates are native to the island and include flora descending south from the Korean Peninsula and flora of tropical and subtropical origins that came from southern Japan. Mt. Hallasan on Jeju Island is covered by temperate evergreen broadleaf forests up to 750 m above sea level, temperate deciduous broadleaf forests between 750 m and 1400 m above sea level, and boreal or subalpine plants from 1400 m to 1950 m above sea level. The top of the mountain features many plants that are boreal or arctic–alpine in nature. Most of these are continental mainland plants that are commonly distributed across Mt. Baekdusan, Manchuria, Siberia, Sino-Himalaya, Mongolia, etc., and, as a result of adaptation by isolation, many endemic plants are also distributed across the top of the mountain [27,28,29].

**Figure 3:**
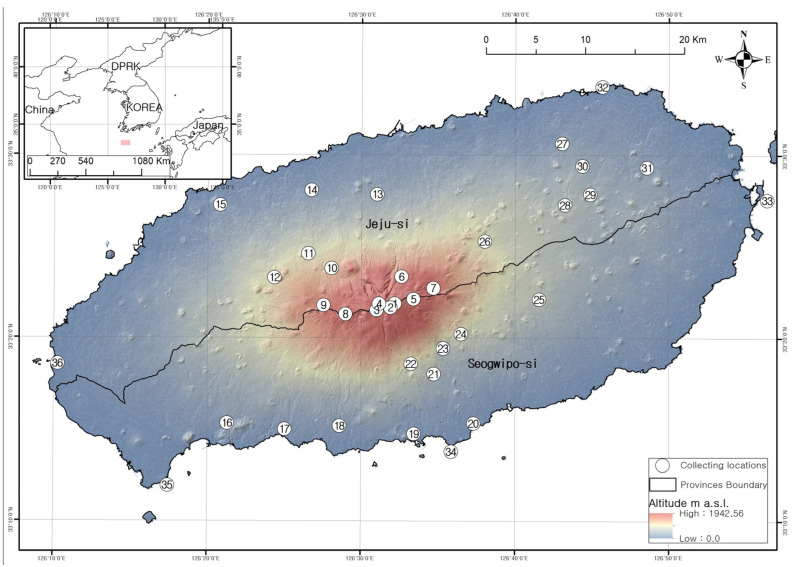
The collection localities are numbered in accordance with the Table 2.

**Figure 4:**
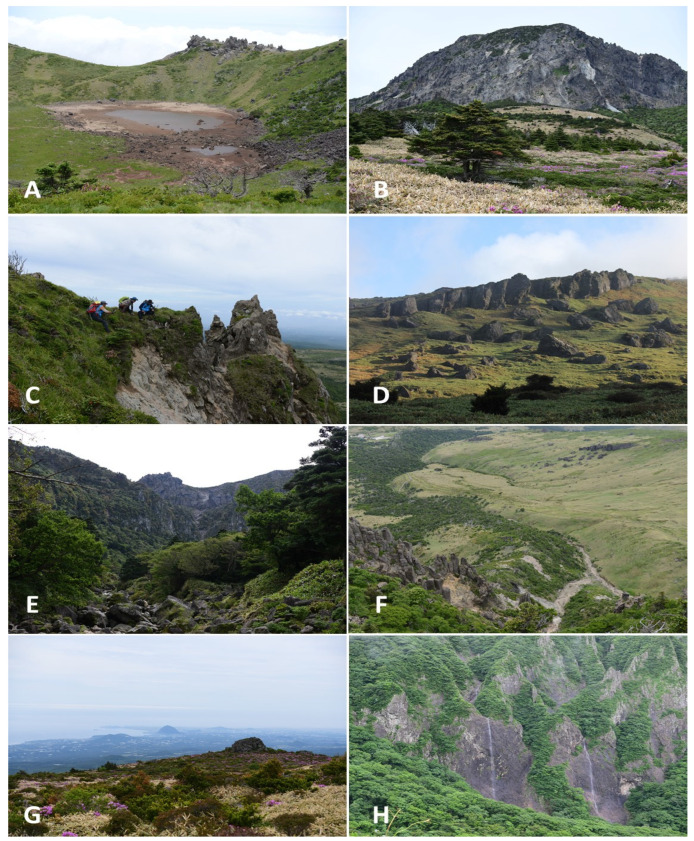
Coniferous forest belt (boreal plant, subalpine forest zone) (alt. 1400–1900 m). (**A**) Inside of Baekrokdam Crater at the top of Hallasan Mountain; (**B**) Outside of Baekrokdam Crater at the top of Hallasan Mountain; (**C**) Trail on Baekrokdam Crater at the top of Hallasan Mountain; (**D**) NW face of Hallasan Mountain; (**E**) Wanggoanreung Valley of Hallasan Mountain; (**F**) Starting point of Tamna Valley in Hallasan Mountain; (**G**) Seonjakgiwat field of Hallasan Mountain; (**H**) Yeongsil Valley of Hallasan Mountain (Photo by S. S Choi, 2010–2022).

**Figure 5:**
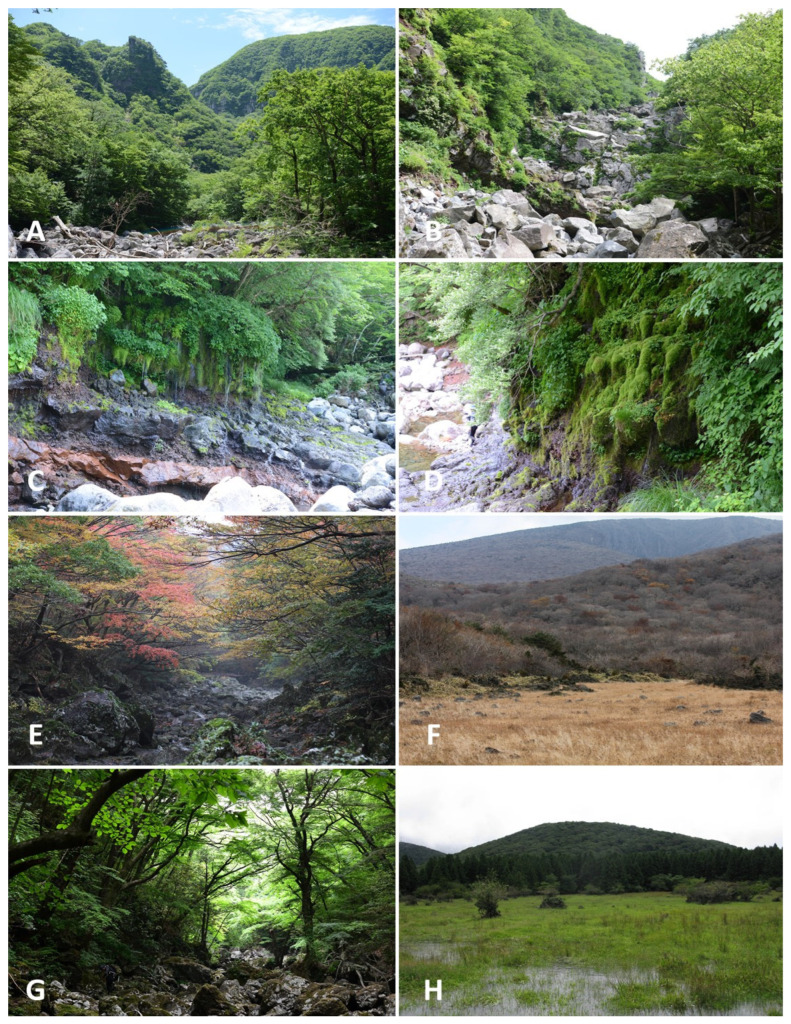
Deciduous broadleaved forest belt (temperate plant zone) (alt. 750–1400 m). (**A**) Middle part of the Y Valley; (**B**) Upper-middle part of the Y Valley; (**C**,**D**) Valley bank of the Y Valley; (**E**) Middle part of the Suak Valley; (**F**) Bolrae Oreum Wetland; (**G**) Middle part of the Tamna Valley; (**H**) Sumeunmulbaengdui Wetland (photo by S. S Choi, 2010–2022).

**Figure 6:**
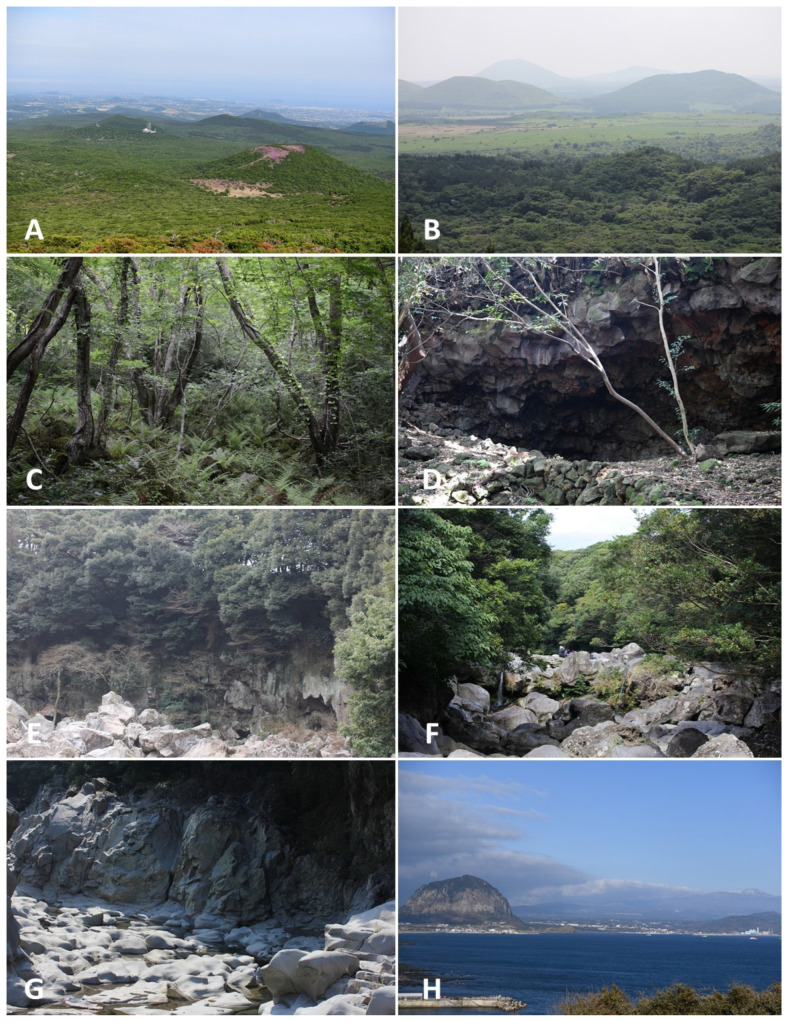
Evergreen broadleaved forest belt (warm temperate, subtropical plant zone) (alt. 0–750 m). (**A**) landscape of Oreum in Seogwipo City; (**B**) Seogeomeun Oreum; (**C**) Gyoraeri Gotjawal; (**D**) Entrance of Woksanjeonggul Cave; (**E**) Low part of Musu Stream; (**F**) Middle part of Hyodong Stream; (**G**) Low part of Hyodon Stream; (**H**) Landscape of seacoast near Sanbangsan (Photo by S. S Choi, 2010–2022).

The typical landscapes and communities where liverworts and hornworts were collected are provided in Table 2 and Figure 3, Figure 4, Figure 5 and Figure 6.

### 4.2. Vegetation

Jeju Island has a variety of plants distributed across its unique natural environment, which includes Hallasan Mountain, large and small oreums, lava caves, and coastal areas. Jeju Island is an area that serves as a stepping stone in Northeast Asia in a biomovement passage to land on the Korean Peninsula and Japan.

In particular, the distribution of vascular plants on Jeju Island differs significantly from that of the land on the Korean Peninsula, occupying a very important position for identifying the distribution of plants entering subtropical regions [23,27]. Jeju Island varies slightly depending on the specific location, but, generally, it can be classified into warm evergreen broadleaved forests up to 750 m above sea level, temperate deciduous broadleaved forests up to 1400 m above sea level, coniferous forests, shrub forests, and subalpine forests from 1400 m to 1950 m above sea level [27,29].

#### 4.2.1. Coniferous Forest Belt (Boreal Plant Zone, Subalpine Forest Zone) (Alt. 1400–1900 m) 

The boreal zone (subalpine zone) on Jeju Island consists of coniferous forests, shrub forests, and subalpine forests with relict plants. The coniferous forests are composed mainly of *Abies koreana* E.H. Wilson, and some *Taxus cuspidata* Siebold and Zucc. The shrub forests are dominated by *Rhododendron mucronulatum* Turcz., *Rhododendron yedoense* f. *poukhanense* (H. Lév.) M. Sugim., *Juniperus chinensis* var. *sargentii* A. Henry, *Sasa quelpaertensis* Nakai, and some *Empetrum nigrum* var. *japonicum* K. Koch growing in the understory. The *Abies koreana* forest is a representative forest of the boreal belt of the Hallasan Mountains on Jeju Island. This forest is distributed from the 1500 m or higher area of Hallasan Mountain to the peak along the valley. The top area (alt. 1800–1900 m) of Hallasan Mountain is mainly covered with rocks and patches of *Diapensia lapponica* var. *obovata* F. Schmidt, *Vaccinium uliginosum* L., *Empetrum nigrum* var. *japonicum* K. Koch, and scattered *Abies koreana*. These plants are species that survived by gradually migrating after the Ice Age to the barren land near the top of Baekrokdam on Hallasan Mountain. These species are currently declining in population and ranges of distribution.

#### 4.2.2. Deciduous Broadleaved Forest Belt (Temperate Plant Zone) (Alt. 750–1400 m)

The vertical distribution of the deciduous broadleaved forest belt is between the evergreen broadleaved forest and the coniferous forest belts, starting at 750 m on the south slope and 550 m on the north slope. As the frequency of evergreen broadleaved trees decreases, areas dominated by *Carpinus tschonoskii* Maxim. and *Quercus serrata* Murray appear, and, as the sea level increases, *Carpinus laxiflora* (Siebold & Zucc.) Blume, *Quercus mongolica* Fisch., *Pinus densiflora* Siebold and Zucc., and *Sasa quelpaertensis* Nakai forest appear. *Pinus thunbergii* Parl., *Pinus densiflora* Siebold and Zucc., and *Cryptomeria japonica* (Thunb. ex L. f.) D. Don afforestation is also distributed in this area.

#### 4.2.3. Evergreen Broadleaved Forest Belt (Warm Temperate, Subtropical Plant Zone) (Alt. 0–750 m)

The warm-temperate evergreen broadleaved forest belt on Jeju Island is distributed from the coast, downstream of the main stream, and on Gotjawal and Oreum, which are areas where unique vegetation occurring only on Jeju Island can be seen. Along the coast, plants such as *Vitex rotundifolia* L. f., *Cyrtomium falcatum* (L. f.) C. Presl, and *Litsea japonica* (Thunb.) Juss. occur. As part of Jeju Island, large and small subsidiary islands such as Munseom Island, Beomseom Island, Seopseom Island, Udo Island, and Chagwido Island are distributed. In particular, Seopseom Island is an uninhabited island, and, although it is small, it is a place where subtropical plants such as the *Castanopsis sieboldii* (Makino) Hatus., *Viburnum odoratissimum* var. *awabuki* (K. Koch) Zabel ex Rümpler, *Elaeocarpus sylvestris* var. *ellipticus* (Thunb.) H. Hara, and ferns such as *Asplenium antiquum* Makino and *Diplazium wichurae* (Mett.) Diels are distributed. From the downstream of the Hyodoncheon and Dosuncheon Streams, which are the main streams of Jeju Island, *Quercus acuta* Thunb., *Camellia japonica* L., *Distylium racemosum* Siebold and Zucc., and *Cleyera japonica* Thunb.), which are warm evergreen broadleaved trees, grow to 750 m above sea level. In various growing environments, such as Gotjwawal, Oreum, and Lava Cave, *Camellia japonica* L., *Daphniphyllum macropodum* Miquel., and *Machilus thunbergii* Siebold and Zucc., etc., are distributed.

### 4.3. Data Analyses

To evaluate the position of the studied flora within the system of local floras in the northern part of the East Asian floristic region, we used detrended correspondence analysis (DCA) based on the matrix completed mainly with the same method used in the identification of the position of bryophyte flora of the southern Kurils [30]. The comparison involves 17 local floras (Table 3) and is based on a matrix where each species was marked as 1 (presence) or 0 (absence). The obtained matrix is available in Table S1, along with the DCA coordinates in a three-dimensional grid. The DCA was visualized in a three-dimensional grid graph, with the third dimension given by a color gradient, as discussed in the Section 2.

## 5. Conclusions

The flora of any mountainous area, especially where there were no ice sheets in the recent geological past, should be taxonomically richer than the flora of plains. However, this phenomenon, due to natural–historical reasons, shows itself in various ways. This statement should be even more true for island regions, where mountain landscapes are surrounded by a sea barrier, which should lead to an effect of insularity. The original purpose of the present account was an attempt to show the originality and richness of the liverwort flora of Jeju-do Island, located near the southern flank of the temperate zone in Pacific Asia. The data on vascular plants showed that, in the lower altitudinal belt, there are species of predominantly subtropical distribution, while, at the mountain apices, the communities with the participation of subarctic species are developed. This promised high taxonomic diversity for liverworts as well. In historical retrospect, starting with U. Faurie at the very beginning of the 20th century, liverworts were collected episodically on Jeju-do Island. A number of liverwort species were described from the Jeju-do as new-for-science. However, the available data, as shown by a simple comparison of species lists, were either incomplete, or, for some reason, the flora of the island turned out to be poorer than other areas of the Republic of Korea. The conducted research has shown the validity of the first assumption. The liverwort and hornwort flora of the studied area turned out to be very rich taxonomically. This richness is determined by the high diversity of landscape elements; the position of Jeju-do Island during the marine regressions, on the path of bilateral floral exchange between Japan and China mainland; and, finally, by sufficiently long periods of island isolation, which allowed a number of rare and disjunctively distributed taxa to survive on the island. The greatest relationships of the liverwort flora of Jeju-do Island was shown with a number of territories in Japan, the central part of the Republic of Korea, and Manchuria. As a real hotspot of taxonomic diversity, the liverwort flora of Jeju-do deserves great attention in conservation of the taxonomic diversity and resource potential of liverworts in East Asia as a whole, as well as organizing monitoring studies of the state of populations of rare taxa.

## Figures and Tables

**Table 1:** Distances between floras in the East Asian Floristic Resion (The degree of proximity of floras, in comparison with the average distance, is highlighted by a color scheme from bright pink for the closest (starting from 12% of the total distance), through intermediate (white) to the most distant (blue, with a maximum of 217% of the total distance).

Abbreviation of Flora	PID	FAL	OLKH	TARD	ITUR	KUN	SHIK	DEO	GAYA	JIRI	TAE	CHAN	SHIR	NH	RISH	VMG	JEJU
PID																	
FAL	27%																
OLKH	87%	62%															
TARD	116%	134%	165%														
ITUR	79%	89%	118%	79%													
KUN	123%	131%	161%	138%	71%												
SHIK	94%	108%	155%	131%	75%	53%											
DEO	55%	69%	129%	150%	108%	122%	77%										
GAYA	77%	64%	94%	189%	130%	140%	114%	68%									
JIRI	62%	43%	70%	170%	111%	131%	110%	72%	26%								
TAE	36%	32%	87%	150%	99%	124%	93%	42%	42%	33%							
CHAN	82%	92%	128%	125%	138%	196%	164%	116%	144%	131%	109%						
SHIR	57%	71%	126%	136%	83%	89%	46%	33%	75%	73%	49%	131%					
NH	134%	159%	220%	151%	148%	152%	105%	103%	170%	173%	142%	159%	106%				
RISH	52%	67%	121%	126%	72%	84%	44%	40%	80%	74%	51%	127%	12%	108%			
VMG	89%	110%	169%	146%	144%	175%	127%	73%	131%	132%	100%	87%	97%	85%	98%		
JEJU	113%	114%	159%	217%	169%	168%	126%	68%	69%	93%	83%	164%	89%	144%	100%	114%	
Average	75%	81%	121%	137%	101%	121%	95%	78%	95%	89%	75%	123%	75%	133%	74%	110%	117%

**Table 2:** The list of collection localities.

Locality	Locality Description	Coordinates	Elevation, m a.s.l.
1	Top of Hallasan Mountain, coniferous forest, *Abies koreana*, *Taxus cuspidata* var. *cuspidata*	33.365281° N 126.5362396° E	1950
2	Northwestern side of Hallasnan Mountain, coniferous forest, *Abies koreana, Diapensia lapponica* var. *obovata*	33.3617278° N 126.531746° E	1856
3	Witse-Orem, grassland, *Empetrum nigrum* var. *japonicum*, *Juniperus chinensis* var. *sargentii*	33.3592515° N 126.5167485° E	1669
4	Upper reaches of Eorimok valley, coniferous forest, *Abies koreana*, *Taxus cuspidata* var. *cuspidata*	33.3652163° N 126.519377° E	1701
5	Shelter of Jindalraebat, coniferous forest, *Abies koreana*, *Sasa quelpaertensis*	33.369404° N 126.5567159° E	1483
6	Upper reaches of Tamla valley, deciduous broadleaved forest, *Carpinus laxiflora* var. *laxiflora*, *Quercus mongolica*	33.3898386° N 126.5436562° E	1168
7	Seongpanak-Sara Oreun, deciduous broadleaved forest, *Carpinus laxiflora* var. *laxiflora*, *Quercus mongolica*	33.3794269° N 126.5782905° E	1117
8	Bolae-Orem, grassland, *Rhododendron yedoense* f. *poukhanense*, *Daphniphyllum teysmannii*	33.35574° N 126.482855° E	1279
9	Sumeunmulbaedyoo wetland, deciduous broadleaved forest, *Quercus serrata*, *Carpinus laxiflora*	33.3637452° N 126.4589471° E	1047
10	Middle reaches of Goangryeongcheon stream, deciduous broadleaved forest, *Carpinus tschonoskii, Daphniphyllum macropodum*	33.3973806° N 126.4678873° E	755
11	Middle reaches of Musucheon stream, evergreen broadleaved forest, *Quercus acuta*	33.4107834° N 126.4425907° E	556
12	Nokkome Oreum, evergreen broadleaved forest, *Castanopsis sieboldii*, *Machilus thunbergii*	33.388749° N 126.4056139° E	621
13	Hancheon valley, evergreen broadleaved forest, *Castanopsis sieboldii*, *Machilus thunbergii*	33.4647953° N 126.5170035° E	194
14	Low reaches of Musucheon stream, evergreen broadleaved forest, *Castanopsis sieboldii*, *Machilus thunbergii*	33.468438° N 126.4458798° E	98
15	Hagamot pond in Aewel, *Nelumbo nucifera*	33.4548485° N 126.3467401° E	68
16	Andeok valley, evergreen broadleaved forest, *Castanopsis sieboldii*, *Machilus thunbergii*	33.2562334° N 126.354811° E	117
17	Cheonjeyeon waterfall, evergreen broadleaved forest, *Castanopsis sieboldii*, *Machilus thunbergii*	33.2509341° N 126.4173409° E	63
18	Dosoncehon valley, evergreen broadleaved forest, *Castanopsis sieboldii*, *Machilus thunbergii*	33.2540595° N 126.4768111° E	87
19	Cheonjiyeon valley, evergreen broadleaved forest, *Castanopsis sieboldii*, *Machilus thunbergii*	33.2467396° N 126.55737° E	44
20	Low part of Hyodoncheon stream, evergreen broadleaved forest, *Castanopsis sieboldii*, *Rhaphiolepis indica* var. *umbellata*	33.2563429° N 126.6220007° E	18
21	Donnaeko valley, evergreen broadleaved forest, *Castanopsis sieboldii*, *Camellia japonica*	33.301331° N 126.5787572° E	301
22	Middle part of Hyodoncheon stream, evergreen broadleaved forest, *Castanopsis sieboldii*, *Camellia japonica*	33.3105832° N 126.5539629° E	530
23	Seondol valley, evergreen broadleaved forest, *Castanopsis sieboldii*, *Camellia japonica*	33.3245784° N 126.5889909° E	374
24	Suak valley, evergreen broadleaved forest, *Quercus acuta*, *Symplocos coreana*	33.3376908° N 126.6083389° E	560
25	Mulyeongari Oreum, evergreen broadleaved forest, *Castanopsis sieboldii*	33.3693219° N 126.6929417° E	482
26	Saryeonisupgil, plantation forest, *Cryptomeria japonica* forest	33.4221289° N 126.6338985° E	561
27	Dongbaekdongsan wetland, evergreen broadleaved forest, *Camellia japonica*, *Quercus acuta*	33.5110923° N 126.7177065° E	124
28	Geomeun Oreum, evergreen broadleaved forest, *Illicium anisatum*, *Aucuba japonica*	33.4551437° N 126.7208625° E	373
29	Cheo Oreum, *Cryptomeria japonica* forest	33.464789° N 126.7481728° E	343
30	Buk Oreum Hammolgu, evergreen broadleaved forest, *Quercus acuta*, *Aucuba japonica* vascular plants	33.4907161° N 126.7396747° E	206
31	Bijarim, *Torreya nucifera* forest	33.4892206° N 126.8096042° E	134
32	Seacoast near Gimnyeong, seacoast, rocks	33.5625669° N 126.7613798° E	1
33	Seongsanilchulbong, cliffs, *Miscanthus sinensis*, *Euonymus japonicus*	33.4593788° N 126.940237° E	134
34	Seopseom Island, evergreen broadleaved forest, *Castanopsis cuspidata* var. *sieboldii*, *Distylium racemosum*	33.2306104° N 126.5978415° E	67
35	Songaksan mountain, Oreum, grassland, *Miscanthus sinensis, Pinus thunbergii*	33.1995314° N 126.29071° E	68
36	Chaguido, seacoast, roadside, *Pittosporum tobira*, *Pinus thunbergii*	33.3097371° N 126.170947° E	74

**Table 3:** The list of local floras involved in the comparison.

No	Abbreviations of Floras	Explanation of the Abbreviation, Literature Sources	Approximate Coordinates
1	**CHAN**	Changbaishan Mts. in north-east China [31,32]	42°00′ N 128°00′ E
2	**DEO**	Deokgyu Mts., Mt. Deogyu National Park, southern part of the Korean Peninsula [33]	36°00′ N 127°30′ E
3	**FAL**	Litovka Mt., Livadysky Range, southern part of the Sikhote-Alin Mts. [34]	43°06′ N 132°47′ E
4	**GAYA**	Gayasan Mts., Gayasan Mountain National Park, southern part of the Korean Peninsula [35]	35°48′ N 128°06′ E
5	**ITUR**	Iturup Island, Kuril Islands [36,37]	45°00′ N 149°00′ E
6	**JIRI**	Jirisan Mts, Jirisan National Park, southern part of the Korean Peninsula [38]	35°20′ N 127°40′ E
7	**KUN**	Kunashir Island, Kuril Islands [36,37,39]	44°00′ N 146°00′ E
8	**NH**	Shiretoko, Nemuro Abashiri Peninsulas, Hokkaido Island [40]	44°00′ N 145°00′ E
9	**OLKH**	Olkhovaya Mt., Alekseevsky Range, southern part of the Sikhote-Alin Mts. ([41], unpublished data)	43°21′ N 133°39′ E
10	**PID**	Livadyskaya Mt., Livadysky Range, southern part of the Sikhote-Alin Mts. ([42], unpublished data)	43°04′ N 132°41′ E
11	**RISH**	Rishiri Island, opposite to western cost of Hokkaido Island [43]	45°00′ N 141°00′ E
12	**SHIK**	Shikotan Island, Kuril Islands [30]	43°30′ N 143°30′ E
13	**SHIR**	Shirakami Mt., Aomori Prefecture, Honshu Island [44]	40°30′ N 140°00′ E
14	**TAE**	Taebaeksan Mts., Taebaeksan Mountain National Park, southern part of the Korean Peninsula [45]	37°06′ N 128°55′ E
15	**TARD**	Tardoki Yani Range, northern part of the Sikhote-Alin Mts. [46]	48°53′ N 138°02′ E
16	**VMG**	East-Manchurian Mts. (Kedrovaya Pad’ Nature Reserve, mountains westward of Razdolnaya River valley, Sinyaya Mt. [47], unpublished data)	43°07′ N 131°28′ E
17	**JEJU**	Jeju Island, the present paper	33°21′ N 126°32′ E

## Data Availability

Not applicable.

## Supplementary Materials

The following supporting information can be downloaded at: https://www.mdpi.com/article/10.3390/plants12122384/s1, Table S1: DCA matrix of taxa distribution in compared floras.
